# Assessing potential reductions of agricultural GHG in countries with different land productivities: Long-term integrated efficiency in DEA hybrid meta-frontier model

**DOI:** 10.1371/journal.pone.0315571

**Published:** 2025-02-04

**Authors:** Bazyli Czyżewski, Łukasz Kryszak

**Affiliations:** Department of Macroeconomics and Agricultural Economics, Institute of Economics, Poznań University of Economics and Business, Poland, Poland; Universita degli Studi del Molise, ITALY

## Abstract

Globally, the agricultural sector is responsible for the emission of ca. 9.3 gigatons of CO_2_ equivalent annually. A realistic efficiency model orientation, considering agricultural policy objectives in a given region of the world, is a crucial premise for finding the optimal path for achieving global targets of emission reduction. The main objective of this article is to assess agricultural greenhouse gases (GHG) potential reductions in countries with different farming productivity accounting for food security and economic performance of agricultural sector. The analysis focuses on non-radial slack that, theoretically, may be easily reduced by the improvement in available resource management. The DEA-based hybrid super-efficiency meta frontier model with undesirable output was employed, using the efficiency approach integrating three sustainability dimensions. The dataset consisted of data from 99 countries (2005–2018) divided into clusters. Several potential model orientations were tested and discussed with regard to agricultural policy objectives. It was found that by reducing slacks, agricultural emissions can be decreased by 0.74 Gt of CO_2_eq per year. Hence, removing only management inefficiencies would help achieve up to 80% of the global reduction targets in agriculture without a substantial technological change. However, the efficiency change component turned out to be mainly negative over the period studied; thus, a specific focus on agricultural policy is needed in terms of supporting farmers with a more rational use of their resources.

## 1. Introduction

Agriculture significantly contributes to climate change. Globally, the agricultural sector has been responsible for 9.3 Gt of CO_2_eq annually when the related land-use change is considered [1] and ca. 5.8 Gt of CO_2_eq when it is not [2].

The problem of agricultural emissions can be largely solved by increasing production efficiency in agriculture. For example, Smith, et al. [3–5] estimated the yearly reduction of agricultural greenhouse gas (GHG) emissions by 0.4 GtCO_2_eq and Havlík, et al. [6] by 0.21 Gt if agrotechnical knowledge is disseminated.

However, it is not clear what the inefficiency slacks are at the global scale. Moreover, the inefficiencies estimation is highly sensitive, firstly, to the adopted model specification in terms of desirable and undesirable outputs, secondly, to assumptions about model orientation.

The specification of the efficiency model is related to the definition of sustainable agriculture adopted and the choice of externalities caused by the agricultural sector to be addressed by the model [7]. The potential answer to this concern can be an integrated approach to efficiency measurement that takes into account holistic definition of Sustainable Food and Agriculture by FAO [8]. To the best of authors’ knowledge, the integrated approach, which takes into account social and environmental issues, has not been used in cross-country efficiency analyses. In other works that deal with multiple-country comparisons of efficiency, environmental issues regarding economic activity are often ignored [9, 10], while in others, the social aspects are not explicitly studied [11].

The choice of the orientation of the efficiency model should fit not only into the theoretical framework but also into the reality of the implemented agricultural policy. The orientation is followed by the choice of mitigation levers. For example, increases in crop and livestock productivity mean output orientation, while constraints on agricultural land expansion or decreases in post-harvest losses are an example of input orientation. Thus, there is often a clash between models’ assumptions and practice. Predominantly, the input orientation has been adopted in eco-efficiency models, as it follows in part from the definition of the eco-efficiency concept: this concept is defined as achieving economic objectives in an environmentally responsible way [12]. The definitions highlight the importance of decreasing environmental impacts, which is interpreted usually as the intention to decrease input use. We argue that it might be an invalid approach bearing in mind the evolution of main agricultural policies that has taken place up to date. The input orientation has been adopted in many articles, including highly cited papers by Picazo-Tadeo, et al. [13], Coelli, et al. [14], or Bonfiglio, et al. [15].

The aim of this article is therefore twofold: i) to assess agricultural GHG potential reductions in countries with different farming productivities (99 countries divided into three clusters, 2005–2018, FAOSTAT data) using an integrated approach that refers to FAO’s concept of Sustainable Food and Agriculture; ii) to prove how orientation matters while discussing agricultural policies of leading food producers in the world.

Therefore, this article adds to the discussion on how improving managerial efficiency contributes to global GHG mitigation targets while taking into account agricultural policies orientation and one of the most important social aspects, namely, food security (FS). The focus is on non-radial slacks, assuming that they can be eliminated without affecting other inputs/outputs under an available technology (see Section 3 for more details).

For the methodological contribution, the DEA-based hybrid super-efficiency meta frontier model with undesirable output is employed and the estimations for different orientations (input-, output- and non-oriented) are compared. The Malmquist Indices under a sequential approach and the Technology Gap Ratio with total factor productivity decomposition are also calculated. Moreover, a rarely adopted hybrid approach with an original recalculation of non-radial slacks is used.

The remainder of the article is structured as follows: in the next section, the theoretical framework for measuring inefficiency slacks is presented and current agricultural policies in the cluster-leading countries (EU, USA, China) are discussed based on the literature review; section 3 is devoted to the presentation of data and methods. Then, in two separate sections, the analysis of the results and discussion of their implications is provided. Finally, conclusions are drawn.

## 2. Theoretical framework for measurement of inefficiency slacks and agricultural policies review in cluster-leading countries

### 2.1 Measuring slacks in terms of GHG mitigation strategies

An effective GHG mitigation strategy needs an adequate diagnosis of the inefficiencies under the current technology. It is easier to improve resource management under the available technology than to develop new technology. The integrated efficiency analysis, which takes into account environmental and social issues, can be a diagnostic tool to assess the room for improvement in a given context (i.e. an accessible technological frontier).

**Fig 1:**
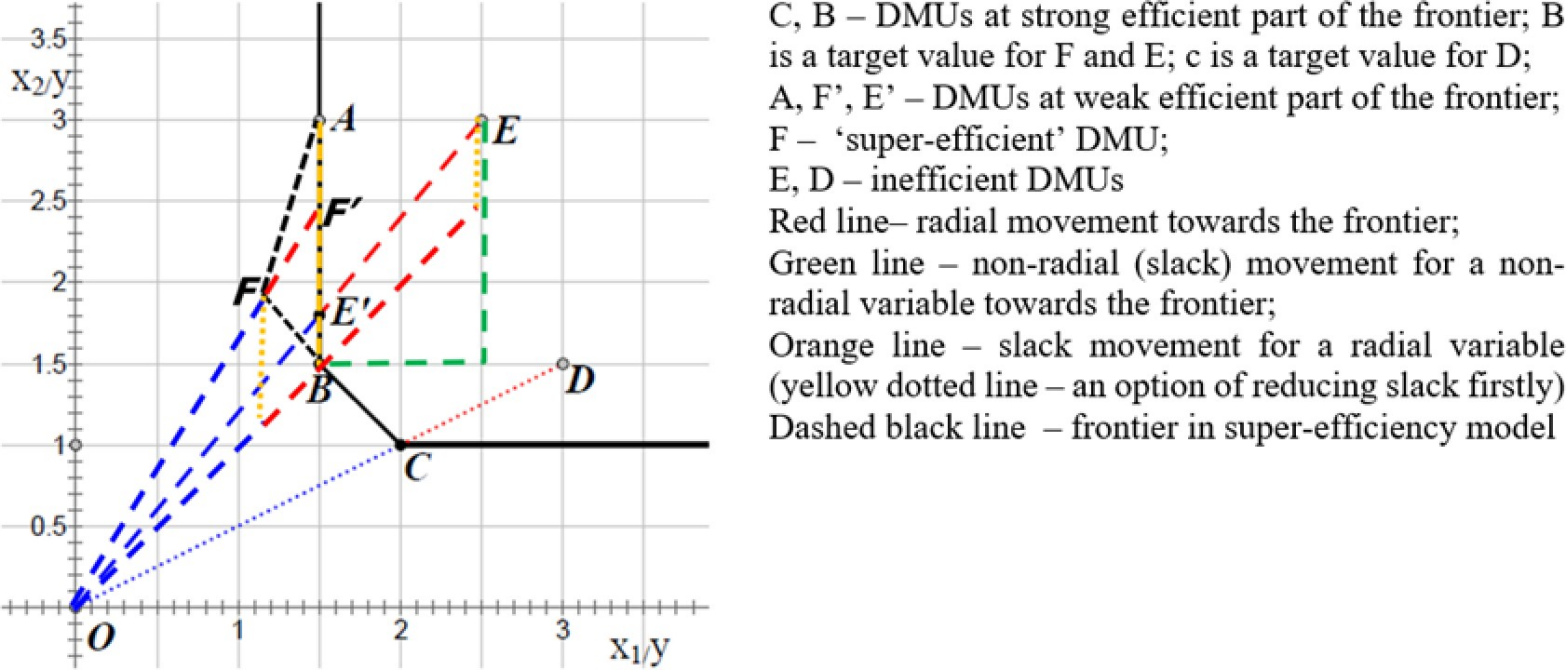
Illustrative examples of slack movement and proportionate movement in the two-inputs model, including the super-efficiency approach; the non-radial slacks are indicated in yellow. Source: Own elaboration based on Cheng [16].

There are two types of inefficiencies in frontier approaches: proportionate (radial) movement and slack (non-radial) movement. The proportionate movement towards a frontier means that all inputs/outputs change by the same percentage. However, this version of inefficiency (sometimes called ‘radial slack’) can be difficult to overcome in practice as, for example, reducing all inputs simultaneously in the input-oriented mitigation strategies seems unrealistic. Thus, the inefficiency slack estimated as non-proportionate (non-radial) movement, which indicates such a change in a specific input/output that does not involve a change in any other input/output, can be more useful [16]. Slack, defined in this way, can also apply to fully efficient units at the technological frontier (see examples in Fig 1).

What does it mean in economic reality, and how is it that GHG reduction is possible without affecting other inputs/outputs? Efficiency analyses in the agricultural sector generally consider inputs such as land, labour, fertilisers, pesticides, energy, livestock and machinery, assuming that they are also an approximant (derivative) of other inputs, such as seeds or seedlings. In studies at the sectoral level, it can be assumed that it is possible to reallocate these inputs between farm groups in such a way as to contribute to a decrease in total agricultural emissions while maintaining the existing quantities/values of inputs. In practice, this could mean increasing the intensity of farming on the most productive farms and implementing sustainable practices (e.g. carbon farming, organic farming, afforestation, etc.) on medium- and low-yielding lands that will reduce emissions and provide more environmental public goods.

At the farm level, it can be assumed that input allocation will be made more eco-effective by introducing more sustainable and precise tillage or animal husbandry techniques, which do not necessarily mean changing the quantity/value of inputs but improving their quality or dosage (e.g. switching from chemical pesticides and mineral fertilisers to organic ones, introducing intercrops, catch crops or precision farming). Thus, it is a matter of knowledge and overcoming the cognitive barriers of farmers. In this regard, the agricultural policy, which directly encourages farmers to adopt sustainable practices, is helpful.

Therefore, in this article, the focus is exclusively on non-radial slack, as indicated by the yellow dotted lines in Fig 1, calling it ‘slack movement’ or ‘real slack’ (RS).

**Fig 2:**
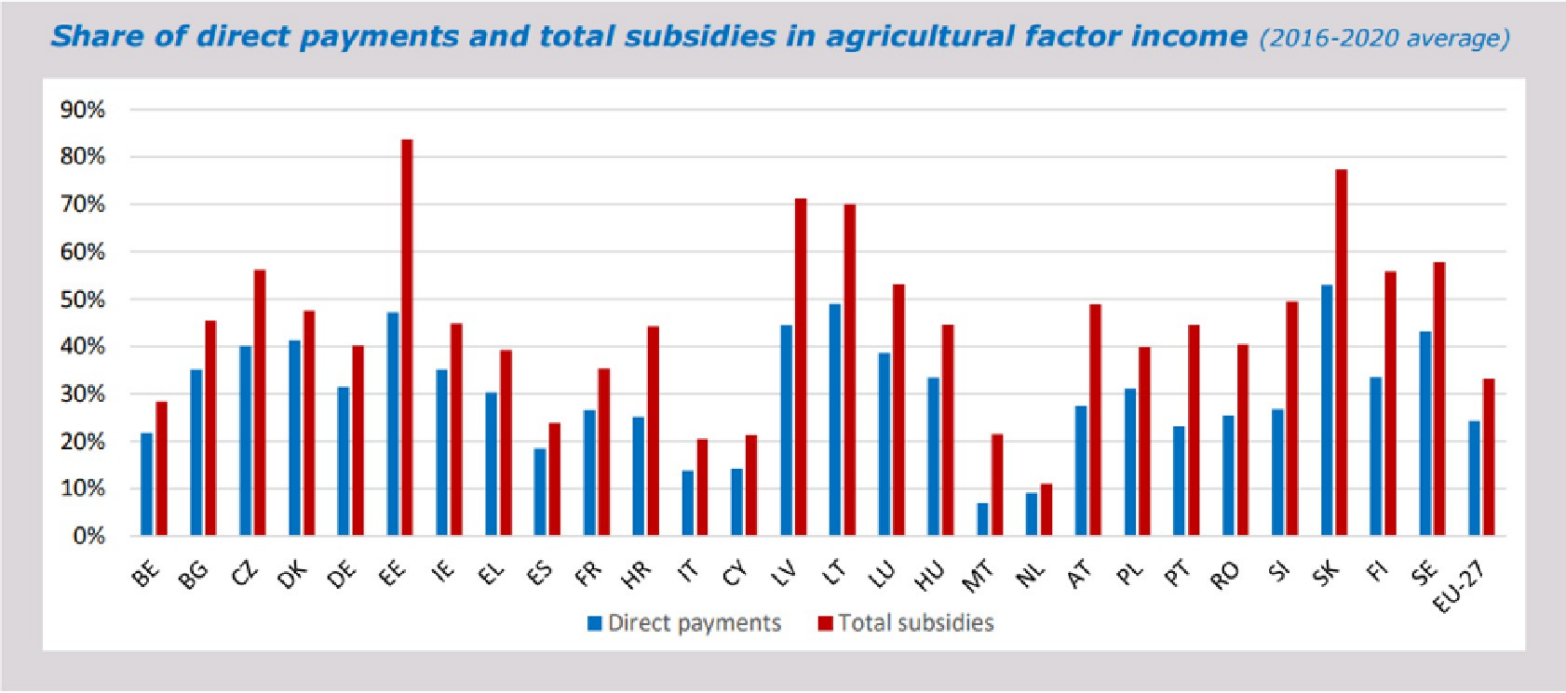
Share of subsidies in income in EU countries. Source: [25].

However, the slacks generated for radial (SRV) and non-radial variables (SNR) are hardly comparable if SRV is calculated as the residual value, which is the case in most software. Thus, if this order were reversed, the percentage of proportionate movement would change after first eliminating SRV from the initial data (the proportionate movement assumes the change of all inputs/outputs by the same percentage). In Fig 2, it is visible that the option of initially reducing a slack movement–the yellow dotted lines–implies a bit longer movement (compared with the slack as a residual shift). To enable comparability of SRV with SNR and to be in line with the adopted definition of slack movement (i.e. such a change in output/input that does not affect any other outputs/inputs), the following formula was used to recalculate RS [17]:

$$\frac{PM}{CL}=\frac{PM+SRV-RS}{CL+RS}$$

After transformation:

$$RS=\frac{SRV*CL}{PM+CL}$$

where *PM* indicates proportionate movement, *SRV* is the original slack movement value, and *CL* is the current level of input or output.

After using this recalculation, removing *RS* from the initial data does not cause any changes in the estimated percentage of the proportionate movement, which is consistent with the assumed definition of slack movement and corresponds to the yellow dotted lines in Fig 1.

The issues of technical, energy and environmental efficiency in agricultural sector are important since the efficiency of a given agricultural ecosystem influence the level of environmental pollutions, including carbon dioxide emission [18, 19]. These efficiencies are also often studied using stochastic frontier analysis (SFA) [20–22]. The advantage of SFA over the DEA is that it allows to decompose the error term into random noise component and inefficiency component. In more advanced approaches of SFA [23] it is possible to decompose the error term into four components when dealing with panel data: time-invariant efficiency, time-variant efficiency, a random shock and units heterogeneity measured by unit individual effect. However, in SFA it is not possible to derive the slack values which are the main interest in this study. In other words, in SFA one can precisely estimate the (in)efficiency level and then investigate the external determinants of the inefficiency levels. However, it is not possible to directly investigate which particular input is used excessively and can be reduced by a given amount or which particular output could be expanded and by how much. For these reasons, the DEA-based approach seems to be more suitable in this study.

### 2.2 Policy orientation review

In this section, the authors ask whether the input-oriented eco-efficiency approach is actually a realistic assumption in the context of countries forging global policies. As the literature review indicates, the input-oriented approach can be justified by the market conditions of agricultural production, which makes farmers as price-takers largely condemned to global commodity prices. According to this view, agricultural commodity prices are shaped globally, and the only thing an individual farmer can do is optimise production factor management. However, the question arises about whether this principle is equally applicable to countries with limited state intervention in agriculture and countries with extensive agricultural support policies. Moreover, from a global perspective and from the point of view of highly developed countries, the orientation towards minimising environmentally harmful inputs while maintaining a given level of production seems rational given the sufficient satisfaction of food needs or even a surplus in this regard. However, the issue looks different from the perspective of less developed countries where FS is under threat.

It is clear that the top economies are creating certain patterns that are setting global emission reduction targets. Agricultural sectors in the EU, the USA and the Peoples Republic of China account for more than 45% of the global agricultural output and manage almost 30% of global arable land. Hence, their leading role in setting global targets for agricultural development and reducing agricultural emissions cannot be overestimated. However, one can point to a number of premises supporting the thesis that the assumption of input-oriented strategies in agricultural sectors of leading countries (that create technological frontier) is unrealistic because agricultural policy largely and sometimes primarily promotes outputs.

In the case of the EU, initially, the explicit aim of the common agricultural policy CAP was to stimulate food production to ensure food safety for EU citizens–a typical production orientation. Meanwhile, an emphasis has been put on increasing and stabilising farmers’ income by market price support.

MacSharry’s reform in 1992, followed by Agenda 2000 and Fischler’s reform in 2003, led to gradual decoupling (breaking the link between subsidies and production) but with historic levels of support in Western European countries that guaranteed high levels of FS.

The most important part of the EU Green Deal, namely, the new Farm to Fork (F2F) strategy, aims to make food production climate-neutral and simultaneously ensures that food is affordable [24].

It is debatable to speak of an orientation towards minimising inputs if the share of subsidies in agricultural income in EU countries averages about 25% for direct payments and about 35% for total subsidies, while in some countries, it exceeds 60% (Fig 2) [25]. In such a situation, the marginal cost curve is artificially lowered, which obviously translates into a higher level of optimal production. Without subsidies, agricultural production in many EU countries would be unprofitable.

Finally, the EU Green Deal announced in December 2019 focuses on achieving the overarching goal of reducing agricultural GHG emissions by 55% (by 2030) compared with the 1990 level. It is worth noting that GHG in this strategy is treated as a kind of aggregate measure of undesirable output and not an input.

Although successive CAP reforms have changed the nature of price interventions, intervention prices are still treated as a ‘safety net‘ to be used in the case of market disruptions. The mechanism is pro-supply, taking the risk of overproduction off the farmers’ shoulders to some extent.

Agricultural insurance subsidies have a similar effect on the optimisation assumption adopted by farmers of the EU member countries as direct payments. Subsidies for less-favoured areas keep land with lower production potential in production, offsetting the higher costs associated with cultivating it.

To sum up, the EU CAP has a number of instruments that strengthen the decoupling introduced by the 2003 Luxembourg reform (the Single area payment scheme and Basic payment system, as well as the modulation mechanisms, i.e. a decrease in support as the area exceeds a certain size). However, at the same time, several solutions indirectly support production and FS, with an emphasis on reducing agricultural GHG emissions as the undesirable output of farming.

In the case of the US, the way the sector is subsidised differs compared to EU countries. However, it is even more visible that the input-orientation assumption sounds unrealistic in these conditions. This opinion can be supported by the following premises that advocate output (or revenue) orientation.

The main goal of the agricultural policy in the USA can be described as ‘decreasing risk, stabilising incomes and increasing farmer’s revenue’ [26].

The agricultural policy in the US is forged by the so-called ‘farm bills’. The most recent Act came into force in 2018 (extended in 2023), reinforcing measures implemented in 2014. To understand the logic of the US agricultural policy, two reforms (from 1996 and 2014) are crucial. Until the 1996 farm bill, farmers obtained payments if the market price of a given agricultural product was lower than a ‘target price’ [27]. The 1996 reform introduced direct payments independent of prices calculated on the basis of historical yields.

In 2008, a revenue-supporting programme called Average Crop Revenue Election (ACRE) was introduced in addition to direct payment [27].

The Farm Bill enacted in 2014 ended the era of direct payments, replacing them with two income-supporting programmes: the Price Loss Coverage Programme (PLC) and Agricultural Risk Coverage Programme (ARC). Under PLC, farmers received payment even if market prices were quite high, whereas in ARC, farmers received payment only if the prices dropped below the reference level (for details [17]). Hence, both programmes reduce the risks related to market conditions and make farmers’ decisions easier.

When it comes to agri-environmental aspects in the USA, they are traditionally supported by the Conservation Reserve Programme (CRP). The programme pays compensation for the diversion of agricultural land in non-crop uses, taking into account the fertility of the land; only less fertile parcels can be sacrificed for environmental purposes. Thus, an increase in the average productivity of agricultural land by eliminating the less fertile ones is encouraged.

Finally, the highest share of spending in the current US farm bill (around 80%) is attributed to the Supplemental Nutrition Assistance Programme to ensure the FS of low-income families [28].

The above milestones of the US agriculture policy clearly indicate that the output orientation would be a more appropriate assumption for efficiency analysis in such a case.

In the case of China’s agricultural policy, it can be said that it is mostly focused on maximising production effects of all the countries analysed, attaching the least importance to the level of inputs (including mainly fertilisation). It is expressed in the following aspects (for details [17]).

China’s agricultural policy tends to achieve three major goals: i) increasing and stabilising farmers’ income, ii) ensuring FS for a growing population and iii) increasing the average farm size [29].

Starting from the 1990s, mass migration to cities began in China. Thus, agricultural policy has focused on output growth by subsidising fertilisers and other polluting inputs.

Agricultural subsidies have been focused mainly on market price support (MPS) as a measure for reducing the income gap between farmers and workers in other sectors; minimum prices on commodities and intervention stocks have been implemented. Since 2015, MPS for crops (except rice and wheat) has been replaced by direct agricultural support and protection subsidies.

As for agri-environmental programmes, the most popular is the ‘Grain for Green’ scheme, which resembles the US CRP while encouraging farmers to convert degraded or abandoned arable land to forest or grassland [30].

To sum up, China’s agricultural policy is oriented mainly towards economic and social goals.

In the context of the above agricultural policy systems, the assumption of input orientation seems unrealistic, as all of these solutions either support agricultural production to some degree or focus on its non-productive effects (e.g. FS, GHG emissions). Despite agricultural policy measures differing around the world, their common point (also in countries other than those discussed above) is moving farmers’ focus from the agricultural inputs to the output side while reducing the effects of market competition.

Last but not least issue concerns the question how do global policy targets correspond with national/regional goals? This issue is particularly problematic because of emission leakage. For example, lower emissions from agricultural production in the EU may be achieved when imports from non-EU countries increase and exports outside the EU decrease [31], even if the EU consumption does not change substantially. The national/regional optimal GHG reduction targets in the agricultural sector depend on the approach to the effort-sharing used. Richards, et al. [32] proposed calculations based on responsibility, capability, equality, responsibility-capability-need and equal cumulative per capita emissions. The approach used in this article is different since the analysis is not about how much countries should decrease their agricultural emission but rather what is possibly relatively easy to achieve.

To sum up, the discussion about production orientation in agriculture is still open, and models for all three possible options are presented. However, it is essential to point out that the choice of orientation is crucial in agricultural efficiency analyses.

## 3. Methodology

### 3.1 Clustering countries with different agriculture productivities

For a cross-country efficiency analysis, the search was for groups of agricultural sectors (DMUs) that are internally consistent but maximally different from each other.

The EU can be treated as a single group, given that the CAP is operating in the EU countries. To create further groups, a cluster analysis of 79 countries from FAOSTATfor which consistent time series are available was conducted (typical island countries were excluded).

It is assumed that the productivity of factors used in agriculture (i.e. relations between agricultural input and output) can be a good indicator to create groups of countries that fulfil the initial condition. Hence, the so-called partial productivity coefficients presented in Table 1 are calculated, using the approach proposed by Czyżewski & Kryszak [17]. However, different models specification and assumptions than in the quoted book are argued here.

**Table 1:** Descriptive statistics: Integrated efficiency variables and factor productivity.

Output/input data in efficiency analysis	Cluster 1		Cluster 2		Cluster 3 (EU)	
	mean	SD	mean	SD	mean	SD
Production (constant bln of international US$, radial)	60	154	24.6	59.3	13.8	16.9
Food security—dietary energy supply adequacy index (percent, *non-radial*)	128	13	117	13	131	11
GHG (CO_2_ equivalent, gigagrams, *radial*)	64677	142075	60193	130995	16748	20089
Land (in 1000 of ha, *radial*)	63,491	124,835	45,792	76,694	7,558	8,350
LSU (mln of standard livestock units, *radial*)	21	53.8	18.1	37	5.31	6.53
Employment (in 1000 of employees, *radial*)	13.065	48.763	9.818	32.774	410	591
Energy (in terajoule, *radial*)	162998	335746	56893	141442	44968	57800
Nitrogen (in mln of tons, *radial*)	1.953	5.724	0.764	2.477	0.393	0.525
Pesticides (in tonnes, *radial*)	99,165	323,696	16,996	51,534	13,066	20,282
**Input productivity (used for clustering): Gross Production Value (current thousand US$) / unit of input**						
Land (ha)	3.607	6.283	0.852	1.389	2.600	2.365
LSU (units)	4.02	2.01	1.19	0.68	2.95	1.03
Employment (employee)	78.619	245.152	11.569	23.754	52.455	41.352
Energy (per terajoule)	7030	29980	12095	49954	404	229
Nitrogen (per tonne)	100	91	135	295	39	24
Pesticides (per tonne)	7758	22450	18856	55069	1461	835

Source

data selection based on the approach by Czyżewski & Kryszak [17].

The *k*-mean clustering procedure with the segment option that specifies *k* nearly equal partitions to be formed was run. Afterwards, the best clustering was chosen according to the Harabasz-Calinski criterion. The division was also tested by multivariate tests of means.

The procedure generated two groups of 29 and 50 countries, as depicted in Table 1. The agricultural sector in Cluster 1 is featured by the highest level of land, livestock and employment productivity and encompasses the most productive agricultural sectors in the world (i.e. the US and China).

Cluster 2 leads in energy and pesticide productivity but mainly due to the low use of these production means. This cluster includes several developing countries with fragmented agriculture (e.g. India, Indonesia) that are key to global FS.

Cluster 3 (the EU) is characterised by the highest input intensity because of the relative scarcity of land in Europe. The average productivity of LSU, employment and land in the EU are significantly lower than in Cluster 1 but higher compared with Cluster 2.

### 3.2 Integrated efficiency model for Sustainable Food and Agriculture

The 99 countries (data for 2005–2018) were divided into three clusters according to agricultural factor productivity, as described in the previous section. All data were obtained from FAOSTAT.

A socially and environmentally adjusted production function (APF) under the DEA-based approach is specified via hybrid **super-efficiency meta frontier model** that allows to calculate Malmquist Indices (MI-sequential) and Technology Gap Ratio with TFP decomposition into efficiency change (EC), technical change (TC), output-based technological change (OBCT), input-based technological change (IBTC) and mixed technological change (MATC).

As assumed in the classical production function, production was treated as output and the inputs reflected three factors of production, namely: land, fixed and working capital and labour. Therefore, the selection of variables for the model is in line with the classical approach (CES models—Dudu & Krsitkova [33]) used in the vast majority of APFs within the DEA framework (e.g. Picazo-Tadeo, et al. [13]; Coelli, et al. [14]; Bonfiglio, et al. [15]; Czyżewski & Kryszak [17]). For the additional desirable and undesirable variables (i.e. food security and GHG emissions respectively), the authors refer to the adopted FAO definition of sustainable agriculture [8].

The approach used was called ‘integrated efficiency’, as it includes three types of outputs referring to each sustainable agriculture dimension: economic, social and environmental. Hence, agricultural production value (in constant international US$) is used as the economic output, with the FS aspect representing the social dimension (i.e. the dietary energy supply adequacy index) and agricultural GHG emissions as an undesirable environmental output.

The models are estimated and compared under different optimisation assumptions: input-oriented, non-oriented and output-oriented, putting the emphasis on the latter, as advocated in Section 3. The focus is on non-radial GHG and FS slack (RS), as justified in the previous section. Below, other assumptions considered in the model are discussed.

The **hybrid approach** seems to be the most suitable for this analysis. On the one hand, radial DEA models seem unrealistic in the real world, as they are unlikely to equally improve inputs/outputs in agriculture [34]. On the other hand, a fully non-radial, slack-based model is also controversial due to some agricultural inputs and outputs that are strongly correlated by definition (e.g. arable land and fertiliser use in crop production). Meanwhile, some variables do not have clear relationships with others (e.g. pesticides, labour, FS indicator). This is why a hybrid model is preffered [35] that makes it possible to use both radial and non-radial inputs/outputs.

The variable is defined as radial if the Spearman correlation coefficient between that variable and any other one is significant and higher than 0.5 [36, 37]. Only the FS index turns out to be non-radial in all models; the other variables are strongly correlated.

Agriculture is typically a scale-sensitive activity at the farm level; thus, it is difficult to advocate variable **returns to scale** (VRS) at the sector level [38]. However, taking into account the global climate crises, one should assume that in countries creating a technological frontier, decreasing return to scale should be emphasised [39], given that available land resources and related environmental capacity have already been depleted in the most productive countries. To address this concern, we assume a non-increasing return to scale (NIRS).

As previously stated, countries from different clusters do not have access to the same technology. Therefore, we employ a **meta-frontier** model [40].

Another issue is the weak discriminating power of basic DEA models, especially in cross-country analysis, resulting in an overrepresentation of efficiency scores equal to 1 [40, 41]. To avoid this problem, we use the so-called super-efficiency model in the version recently used by Wang et al. 2019 [37]. This model produces different scores for efficient DMUs (equal to 1 or higher); the higher the score, the better positioned the DMU.

The technical specification of the adopted model is as follows:

Let the input data matrix be $X\in R_{+}^{f\times n}$, where *n* and *f* are the numbers of DMUs and inputs (factors of production), respectively. This input data matrix is decomposed into radial ${(X}^{R}\in R_{+}^{f_{1}\times n})$ and non-radial parts ${(X}^{NR}\in R_{+}^{f_{2}\times n})$. The total number of inputs is equal to *f* = *f*_1_+*f*_2_. There are desirable output and undesirable output matrix: $Y^{g}\in R_{+}^{s\times n}$, and $Y^{b}\in R_{+}^{z\times n}$, where *s* and *z* are respectively the numbers of good and bad outputs. For inputs, these two matrices are also decomposed into radial and non-radial components: $Y^{gR}\in R_{+}^{s_{1}\times n}$, and $Y^{gNR}\in R_{+}^{s_{2}\times n}$ for desirable outputs; $Y^{bR}\in R_{+}^{z_{1}\times n}$ and $Y^{bNR}\in R_{+}^{z_{2}\times n}$ for undesirable outputs. DMUs are clustered into C groups.

For the DMU $(x_{0},y_{0})=(x_{0}^{R},x_{0}^{NR},y_{0}^{gR},y_{0}^{gNR},y_{0}^{bR},y_{0}^{bNR})\in P$ the hybrid super efficiency DEA model under meta frontier is specified as:

$$min\frac{1-\frac{f_{1}}{f}(1-\theta )-\frac{1}{f}\sum_{i=1}^{f_{2}}\frac{s_{i}^{NR-}}{x_{ik}^{NR}}}{1+\frac{s_{1}}{s}(\phi -1)+\frac{1}{s}\sum_{r=1}^{S_{2}}\frac{s_{r}^{NR+}}{y_{rk}^{gNR}}+\frac{z_{1}}{z}(\omega -1)+\frac{1}{z}\sum_{t=1}^{z_{2}}\frac{s_{t}^{NR-}}{y_{tk}^{bNR}}}$$

s.t.


$$\sum_{c=1}^{C}\sum_{j=1,\neq k}^{n}x_{ij}^{R}\lambda_{j}\leq \theta x_{ik}^{R},i=1,2,\ldots ,f_{1}$$

$$\sum_{c=1}^{C}\sum_{j=1,\neq k}^{n}x_{ij}^{NR}\lambda_{j}-s_{i}^{NR-}\leq x_{ik}^{NR},\;i=1,2,\ldots ,f_{2}$$

$$\sum_{c=1}^{C}\sum_{j=1,\neq k}^{n}y_{rj}^{gR}\lambda_{j}\geq \phi y_{rk}^{gR},r=1,2,\ldots ,s_{1}$$

$$\sum_{c=1}^{C}\sum_{j=1,\neq k}^{n}y_{rj}^{gNR}\lambda_{j}+s_{r}^{NR+}\geq y_{rk}^{gNR},r=1,2,\ldots ,s_{2}$$

$$\sum_{c=1}^{C}\sum_{j=1,\neq k}^{n}y_{tj}^{bR}\lambda_{j}\leq \omega y_{tk}^{bR},t=1,2,\ldots ,z_{1}$$

$$\sum_{c=1}^{C}\sum_{j=1,\neq k}^{n}y_{tj}^{bNR}\lambda_{j}-s_{t}^{NR-}\leq y_{tk}^{bNR},t=1,2,\ldots ,z_{2}$$

$$\sum_{c=1}^{C}\sum_{j=1,\neq k}^{n}\lambda_{j}=1,s_{i}^{NR-}\geq 0,s_{r}^{NR+}\geq 0,s_{t}^{NR-}\geq 0$$

$\sum \lambda_{j}\leq 1,j=1,2,\ldots ,n(j\neq k),s_{i}^{NR-}\geq 0,s_{r}^{NR+}\geq 0,s_{t}^{NR-}\geq 0,\lambda \geq 0,\theta \leq 1,\phi \geq 1,\omega \geq 1$, where ∑*λ*_*j*_≤1, means that we assume non-increasing returns to scale and $s_{i}^{NR-}\geq 0,s_{r}^{NR+}\geq 0,s_{t}^{NR-}\geq 0$ are the slacks for non-radial inputs, desirable outputs and undesirable outputs, respectively.

To evaluate the changes in efficiency, the sequential MI is engaged [42]. As a final step, the *metatechnology ratio* was calculated $\frac{\theta_{o}}{\theta_{ok}}$ for the *o* object from the *k-*th group [43]. In the paper by Battese, et al. [44], this quotient is called the technology gap ratio (TGR). Its value is no greater than one, and the closer it is to one, the closer the technology of the *k-*th group to the theoretical common technology of all groups at the data point corresponding to object *o*.

## 4. Results

The descriptive statistics of the sample under study, including the variables and clusters description, are presented in Table 1. Data based on Czyżewski & Kryszak [17].

Adding the agricultural GHG emissions (the current level) from Clusters 1–3 gives 4.91 GtCO_2_eq yearly on average (i.e. 85% of the global emissions from agriculture). In the used sample of countries, inefficiency slack is equal to **0.74 GtCO**_**2**_**eq yearly** in total (0.25 + 0.44 + 0.045), according to calculations in the output-oriented model (Table 2). The detailed information on slacks for single countries in different model orientations are proved in Tables A1-A3 in S1 File.

**Table 2:** The average value of efficiency and slacks on food security and GHG emission in clusters under different model assumption.

Cluster	Median group score	Median meta-frontier score	TGR	FS av. slack	Slack FS %	Current av. level FS	Total Slack GHG (Gt)	Slack GHG in %	Current level GHG(Gt)
Output oriented									
Cluster 1	1.06	1.01	0.94	-2.04	-1.59%	128	-0.25	-13.91%	1.81
Cluster 2	1.04	0.87	0.86	3	2.56%	117	-0.44	-16.53%	2.65
Cluster 3	1.01	0.98	0.98	-1.56	-1.19%	131	-0.045	-10.05%	0.45
Input oriented									
Cluster 1	1.27	1.05	0.88	2.81	2.20%	128	-0.12	-6.45%	1.81
Cluster 2	1.12	0.75	0.69	2	2.10%	117	-0.23	-8.78%	2.65
Cluster 3	1.11	1.01	0.95	0.47	0.36%	131	-0.045	-10.09%	1.81
Non-oriented									
Cluster 1	1.08	1.02	0.95	-1.74	-1.36%	128	-0.19	-10.40%	1.81
Cluster 2	1.06	0.8	0.79	3	2.39%	117	-0.35	-13.19%	2.65
Cluster 3	1.01	0.98	0.97	-0.98	-0.74%	131	-0.048	-10.66%	1.81

Note: The signs for GHG slacks are always negative showing by how much emission should (and could) be reduced. Positive slack values for FS showing by how much FS should be improved. Negative slack values for FS, in turn, shows by how much F could be decreased without depleting efficiency

Source: Own elaboration based on FAOSTATA data.

The results of presented analysis are more optimistic than those of Smith, et al. [3, 4] and Havlík, et al. [6]. However, the cited authors did not study integrated efficiency, which takes into account the three dimensions of sustainable agriculture.

Although it is difficult to compare our findings with the estimations of potential productivity growth, the Food, Agriculture, Biodiversity, Land-use, and Energy report forecasts a growth of crop productivity measured in kcal per ha by 25%–80% and livestock productivity in kcal per livestock unit by 10%–90% (until 2050; 2015 as a baseline) depending on a country profile and argues that the focus on productivity is one of the major mitigation strategies [45]. The Intergovernmental Panel on Climate Change (IPCC) (2018) report estimates that increasing farming efficiency allows for achieving 20% of the required reduction in agricultural emissions.

**Fig 3:**
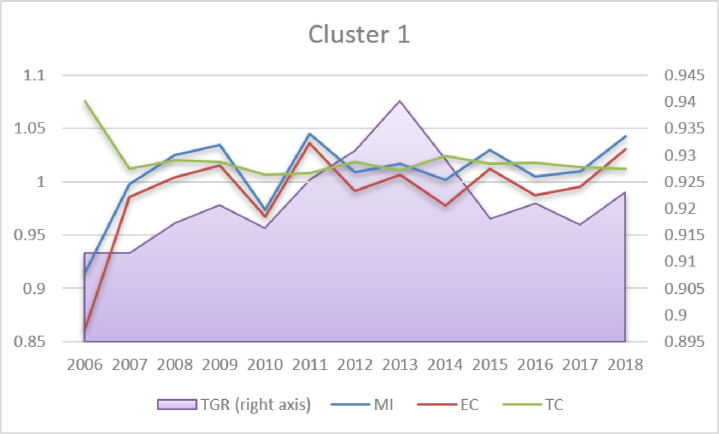
Malmquist index, efficiency change, technological change and technological gap ratio (2006–2018 averages). Source: Own elaboration.

**Fig 4:**
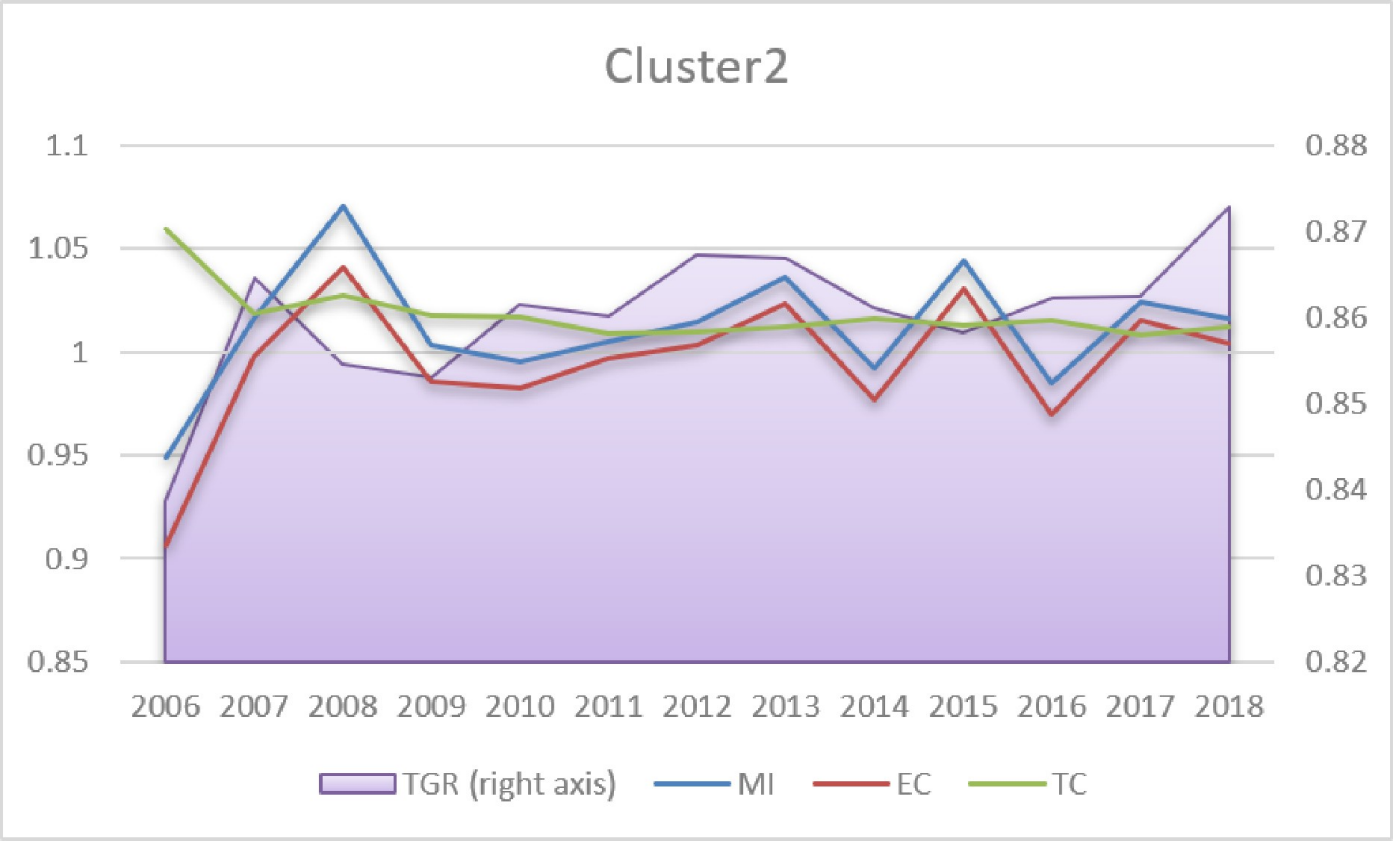
Malmquist index, efficiency change, technological change and technological gap ratio (2006–2018 averages). Source: Own elaboration.

**Fig 5:**
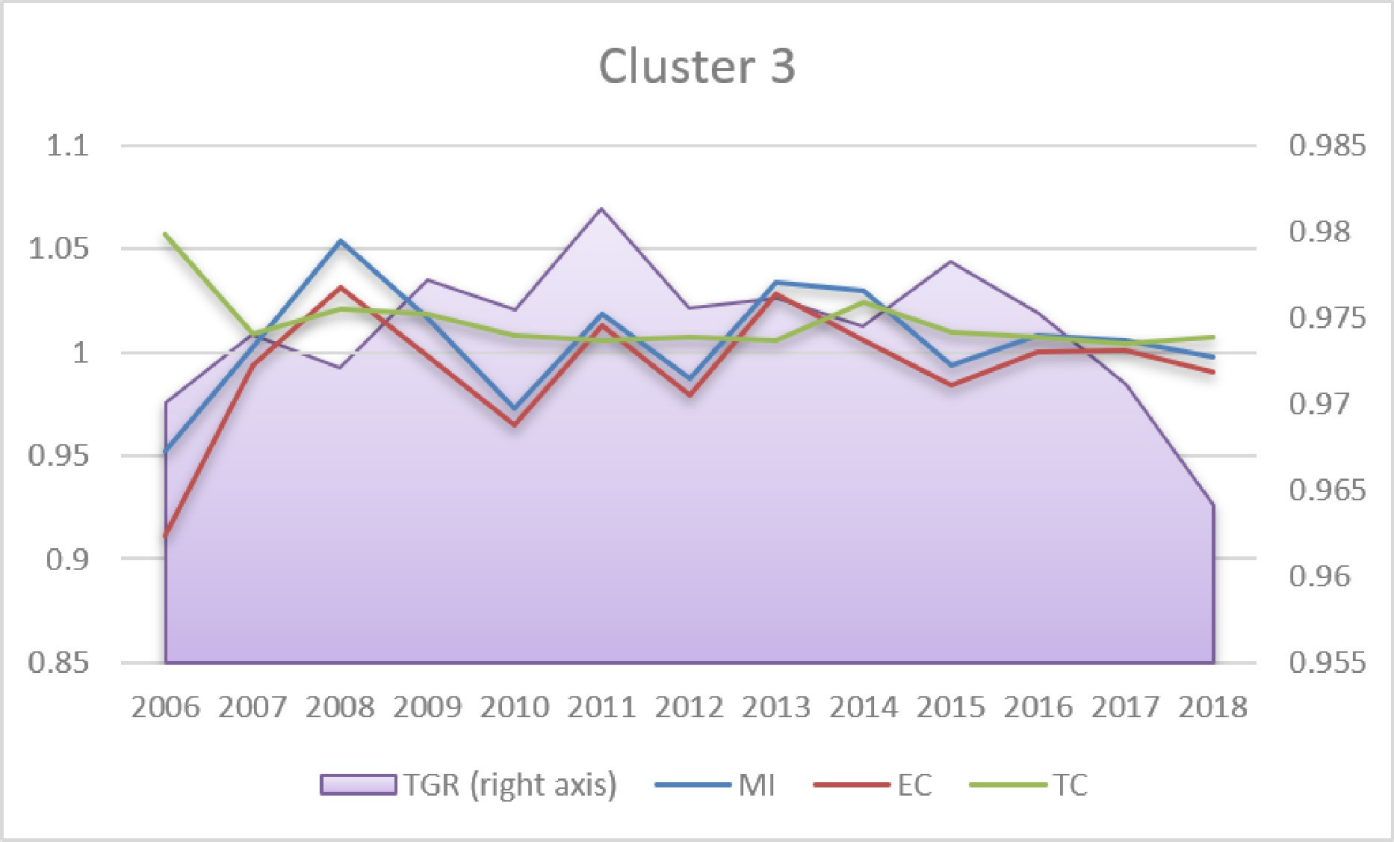
Malmquist index, efficiency change, technological change and technological gap ratio (2006–2018 averages). Source: Own elaboration.

Regarding MI change (Table A4 in S1 File), the estimations are in line with the findings obtained by other authors (e.g. Hoang & Coelli [46]; Eberhardt & Teal [47]), who argued that positive MI in agriculture in the long run results primarily from the TC, whereas EC is negative, as it is the case in all clusters in the present analysis. Analysing the dynamics of MI, TC, EC and TGR (Figs 3–5), it can be seen that long-term MI growth is positive but constant and relatively low (below 1.2%, Table A4 in S1 File). TC is also positive (above 1); however, a slightly decreasing tendency is visible in Clusters 2–3 and stagnation in Cluster 1. The EC dynamics are negative and stable in the majority of periods. The most striking fluctuations concern TGR (left axis in Figs 3–5). Both in Clusters 1 and 3, one can see a reversal of the upward trend in 2013 and 2015, respectively. In Cluster 2 (developing countries), TGR has been slightly increasing in the long run. This finding may be worrisome, as it signifies the slow decline of agricultural technological advantages in most developed countries.

Another important question that arises is: what agricultural policy in the context of GHG mitigation should be considered a global model and followed? While answering this question, the model orientation especially matters. In the input-oriented approach (Table 2), the smallest average GHG inefficiencies (in percentages) are observed in Cluster 1 (–6.45%), followed by Cluster 2 (–8.78%) and, finally, Cluster 3 or the EU (–10.09%). However, the output orientation (advocated in this paper) reverses this order: –10.05% in Cluster 3, –13.91% in Cluster 1 and –16.53% in Cluster 2 (Table 2), that is, the EU strategies yield the best GHG mitigation strategy. In the non-oriented approach, which is a kind of compromise, the results for Clusters 1 and 3 even out (Table 2).

In addition to environmental order, FS inefficiency also matters in integrated efficiency. In the output-oriented model, Clusters 1 and 3 have some surpluses in terms of dietary energy supply (in the case of desirable outputs, a minus sign indicates excess efficiency; in the case of undesirable outputs, such as GHG, a minus means an inefficiency slack). These excess efficiencies are the rationale for introducing additional mitigation levers, such as dietary shifts and decreases in food waste. By contrast, as expected, inefficiencies appear in Cluster 2 in terms of the FS index, averaging 2.56% (Table 2). Although the average levels of the dietary energy adequacy index in all clusters are above 100, indicating sufficient satisfaction of caloric needs, there are countries with indices below 100 in Cluster 2 (Tables A1-A3 in S1 File). In these cases, resolving the FS issue is the priority of agricultural policy, and GHG inefficiencies recede into the background.

## 5. Discussion

At the global scale, agriculture generates ca. 5.8 GtCO_2_eq of GHG emissions yearly [2], accounting for more than 10% of the total emissions from anthropogenic sources. The most problematic is the rapid growth of these emissions which, according to the cited author, will reach 30%–55% by 2030 (which gives 7.5–9.0 GtCO_2_eq yearly). Wollenberg, et al. [2] estimated that we need to reduce agricultural GHG emissions by 0.92–1.37 GtCO_2_eq yearly by 2030 to limit global warming in 2100 to 2 above the pre-industrial levels. If one considers a more demanding scenario of limiting global warming to 1.5° C above the pre-industrial level in 2050 [48], agricultural emissions should then be ca. 1/2 of what they would be under the baseline scenario [49], which assumes 7.19–8.93 GtCO_2_eq of GHG in 2050 (a reduction by 3.6–4.5 GtCO_2_eq per year).

If one assumes that GHG inefficiencies will still exist in the near future (i.e. in 2030, according to the cited baseline scenario), the conclusion is that a vast majority of the required GHG reductions (ca. 80%) can be achieved simply by removing non-radial slack without a substantial technological change. In the more ambitious scenario by Leahy, et al. [49], eliminating GHG non-radial slack attains 16%–20% of this target. However, if the input-oriented model or non-oriented model into account are considered, the estimated inefficiency slack is lower—0.4 GtCO_2_eq and 0.59 GtCO_2_eq, respectively (Table 2). It means that in less demanding scenario by Wollenberg, et al. [2] up to 43.5% of required GHG reductions can be achieved in input-oriented model and up to 64% in non-oriented model. The differences between different model orientations are therefore noticeable. This raises the importance of appropriate model selection.

The world is dominated by small farms. Of the 570 million farms worldwide, 85% or 480 million have 2 hectares or less, being the source of livelihood for almost 50% of the world’s population who live in rural areas [50]. However, it is worth noting that the threshold for a very small farm in the EU accounts for €15,000 of standard output per year, which is a large income in developing countries. Nonetheless, smallholder farms are always the most vulnerable to various cognitive limitations [51] due to lower levels of education, limited access to information, weak ties to the market, bargaining power in the value chain and various path dependencies.

The estimated slack may be a result of these cognitive barriers. Small farms can be more ecologically and socially efficient with the same resources as today. Hence, the results of this paper indicate the need for agricultural policies in different parts of the world to focus on removing cognitive barriers and spreading knowledge about sustainable farming practices, such as through agricultural advisory services aimed primarily at small farms or promoting precision farming.

Several carbon-farming practices can be implemented without large expenditures, contributing to increased food production in the long term with improved carbon sequestration through professional fertilisation plans, adopting crop rotations, intercrops, catch crops, appropriate application of organic fertilisers, mixing straw and other plant residuals in the soil, and so on.

## 6. Conclusions

In this article, the GHG and FS long-term inefficiencies in countries with different farming productivities are assessed. The analysis has shown that efficiency calculations should be preceded by careful consideration of the model assumption. The appropriate choice of model specification, including its orientation, is important not only from a theoretical point of view but can also have a significant impact on the results.

The present study underlines the importance of GHG emissions and FS problems from both environmental and social points of view. It is found that the total slack on GHG in the used sample of countries amounts to 0.74 GtCO_2_eq yearly, while the current emission of GHG in these countries was 4.91 GtCO_2_eq. Therefore, the slack constitutes around 15% of the global emission. In other words, 15% of the global emission can be saved relatively easily if inefficiencies are eliminated. Such a reduction in emissions would bring us much closer to achieving the global objectives.

When it comes to FS, it seems at first glance that it is an important problem only in less-developed countries since some surpluses in this term are visible in the other group of countries. However, it means that agricultural policies in the most developed countries are not optimal. Some of these countries produce too much emission and have negative slacks on FS (which means that FS is achieved with some excess).

The evidence suggests that policies should be reoriented towards support for more directly targeted decarbonisation schemes and dietary shifts. However, it is possible that agri-environmental policies could be more effective if greater emphasis were placed on more rational farm resource management and the reduction of cognitive barriers. In developing countries, the emphasis in politics should be somewhat different, as the priority is to ensure food security, but it does not mean that environmental issues should not be taken into consideration. The analyses have shown that efficiency dynamics and its sources are quite similar between different groups of countries. The progress in efficiency is relatively slow. The exogenous technological change is positive by definition but small, while endogenous efficiency change is often negative. Such results suggest that there are two big challenges facing agriculture on a global scale. First, policies should facilitate the transfer of technology produced outside the sector to agricultural use. Our results indicate the need for agricultural policies in different parts of the world to focus on removing cognitive barriers and spreading knowledge about sustainable farming practices, such as through agricultural advisory services aimed primarily at small farms or promoting precision farming. Second, policies should support farmers in a more rational and knowledge-based use of production means. One potential avenue for achieving this goal is the design of agri-environmental measures that directly encourage the implementation of sustainable farming practices. One such example is the new EU ‘eco-schemes’. However, the success of such measures will depend on the quality of the advisory extension services that are created to support their implementation.

The study has some limitations. One aspects is that due to problems with accessing comparable data, the analysis does not cover all countries in the world (although it comprises the vast majority of global agricultural production). In future studies, the spatial scope can be expanded. Furthermore, the approach presented in this paper can also be adapted on a micro level. In this way, it would be possible to assess the contribution of individual farms to the potential to achieving environmental objectives. In the present study the focus was on efficiency and slacks levels and dynamics. However, in future it will be also valuable to study their determinants to better know which factors can influence the efficiency level.

## Supporting information

S1 File Detailed information on average efficiency scores and slacks for all countries in study under different model assumptions and metafrontier TFP decomposition. (DOCX)

S1 Dataset (XLSX)
