# Supplementary material for: Assessing potential reductions of agricultural GHG in countries with different land productivities: Long-term integrated efficiency in DEA hybrid meta-frontier model
Source: PLoS One. 2025 Feb 4;20(2):e0315571. doi: 10.1371/journal.pone.0315571 (PMC11793737; doi:10.1371/journal.pone.0315571)
Supplement: S1 File — (DOCX) [file pone.0315571.s001.docx]

**Supplementary material**

Table A1. Agricultural GHG and food security non-radial slacks in output-oriented model (O-O); 2005-2018 yearly averages

| **Cluster 1** | **Score** | **MF** | **TGR (MF:SCORE)** | **Slack Fs** | **Slack FS %** | **Current level FS** | **Slack GHG (Gg)** | **Slack GHG %.** | **Current level GHG(Gg)** |
| --- | --- | --- | --- | --- | --- | --- | --- | --- | --- |
| Argentina | 1.37 | 1.26 | 0.94 | -10.21 | -7.72% | 132 | -55308 | -41.98% | 131740 |
| Algeria | 1.24 | 1.11 | 0.94 | -9.91 | -6.99% | 142 | -2058 | -18.44% | 11161 |
| USA | 1.13 | 1.11 | 0.99 | -1.06 | -0.72% | 148 | -46297 | -12.21% | 379283 |
| Egypt | 1.11 | 1.1 | 0.99 | -1.62 | -1.14% | 142 | -4272 | -13.69% | 31194 |
| Ghana | 1.21 | 1.1 | 0.95 | -6.18 | -4.81% | 129 | -1203 | -13.04% | 9224 |
| Malawi | 1.16 | 1.1 | 0.96 | -8.71 | -7.43% | 117 | -840 | -17.49% | 4805 |
| China | 1.07 | 1.07 | 1 | -0.61 | -0.48% | 127 | -74594 | -10.70% | 697231 |
| Israel | 1.21 | 1.07 | 0.95 | -11.4 | -7.31% | 156 | -144 | -10.26% | 1406 |
| Ukraine | 1.09 | 1.07 | 0.99 | 0.9 | 0.71% | 127 | -3781 | -12.67% | 29848 |
| Philippines | 1.07 | 1.06 | 0.99 | -0.29 | -0.25% | 116 | -9737 | -15.35% | 63416 |
| Turkey | 1.07 | 1.05 | 0.99 | -4.2 | -2.73% | 154 | -2055 | -5.02% | 40969 |
| Viet Nam | 1.05 | 1.05 | 1 | -1 | -0.84% | 119 | -7394 | -10.17% | 72725 |
| Russian Federation | 1.04 | 1.04 | 0.99 | -0.07 | -0.05% | 133 | -10154 | -10.36% | 98022 |
| Japan | 1.05 | 1.02 | 0.97 | 0 | 0.00% | 112 | -2612 | -10.79% | 24207 |
| Thailand | 1.05 | 1 | 0.95 | 0.18 | 0.16% | 113 | -16257 | -21.08% | 77133 |
| North Macedonia | 1.24 | 0.99 | 0.84 | -17.77 | -14.93% | 119 | -261 | -18.36% | 1421 |
| Chile | 1.03 | 0.96 | 0.94 | 3.48 | 2.83% | 123 | -940 | -7.94% | 11845 |
| Côte d'Ivoire | 1.04 | 0.96 | 0.92 | 0.01 | 0.01% | 128 | -454 | -7.10% | 6394 |
| Malaysia | 1.03 | 0.95 | 0.92 | 6.93 | 5.72% | 121 | -1542 | -10.86% | 14197 |
| Canada | 0.94 | 0.92 | 0.98 | 15.12 | 10.92% | 139 | -5012 | -8.81% | 56924 |
| Mozambique | 1.04 | 0.91 | 0.94 | 4.55 | 4.42% | 103 | -3531 | -19.51% | 18095 |
| Norway | 1.07 | 0.85 | 0.8 | -9.63 | -7.10% | 136 | -812 | -15.80% | 5139 |
| Switzerland | 1.05 | 0.85 | 0.81 | -0.44 | -0.33% | 133 | -766 | -12.67% | 6048 |
| Albania | 1.02 | 0.79 | 0.78 | -4.95 | -3.78% | 131 | -576 | -17.27% | 3337 |
| Jordan | 1.16 | 0.79 | 0.7 | -12.74 | -10.51% | 121 | -169 | -15.47% | 1092 |
| Saudi Arabia | 0.83 | 0.75 | 0.91 | 16.09 | 12.10% | 133 | 0 | 0.00% | 5841 |
| Sri Lanka | 1.04 | 0.7 | 0.67 | -0.25 | -0.22% | 112 | -1146 | -17.20% | 6663 |
| Armenia | 1.01 | 0.67 | 0.67 | -3.37 | -2.80% | 120 | -5 | -0.30% | 1586 |
| **Mean/Median (for Scores)** | 1.06 | 1.01 | 0.94 | -2.04 | **-1.59%** | 128 | -8997.16 | **-13.91%** | 64677 |
| **Total** | - | - | - | - | **-** | - | **-251 920** | **-** | **1 810 946** |
| **Cluster 2** | **Score** | **MF** | **TGR (MF:SCORE)** | **Slack FS** | **Slack FS %** | **Current level FS** | **Slack GHG** | **Slack GHG %.** | **Current level GHG** |
| Brunei Darussalam | 1.67 | 1.29 | 0.9 | -27.29 | -21.43% | 127 | -31 | -27.28% | 115 |
| Niger | 1.24 | 1.23 | 0.99 | -4.34 | -3.64% | 119 | -7278 | -29.98% | 24277 |
| Indonesia | 1.2 | 1.19 | 1 | -2.1 | -1.78% | 118 | -36177 | -21.89% | 165286 |
| Iceland | 1.18 | 1.14 | 0.97 | -8.95 | -6.54% | 137 | -170 | -26.53% | 640 |
| India | 1.14 | 1.13 | 1 | -0.4 | -0.38% | 106 | -134995 | -18.55% | 727678 |
| Pakistan | 1.15 | 1.13 | 0.99 | 0 | 0.00% | 107 | -55056 | -33.44% | 164626 |
| Iran (Islamic Republic of) | 1.11 | 1.09 | 0.98 | -0.13 | -0.10% | 127 | -4912 | -12.93% | 37981 |
| Nepal | 1.14 | 1.09 | 0.97 | -2.05 | -1.73% | 119 | -3702 | -14.61% | 25339 |
| Cameroon | 1.1 | 1.08 | 0.98 | -1.7 | -1.43% | 118 | -1414 | -10.91% | 12963 |
| Brazil | 1.11 | 1.06 | 0.96 | -0.57 | -0.43% | 132 | -85367 | -16.86% | 506303 |
| New Zealand | 1.1 | 1.04 | 0.97 | -0.27 | -0.21% | 127 | -6034 | -13.62% | 44318 |
| Kazakhstan | 1.11 | 0.99 | 0.9 | -2.11 | -1.63% | 130 | -4632 | -18.91% | 24497 |
| Ethiopia | 1.01 | 0.98 | 0.97 | -0.66 | -0.65% | 101 | -10278 | -10.38% | 99013 |
| Tunisia | 1.06 | 0.97 | 0.93 | -3.17 | -2.23% | 142 | -76 | -1.64% | 4633 |
| Australia | 1.08 | 0.93 | 0.87 | -1.75 | -1.32% | 132 | -22350 | -13.98% | 159919 |
| Kenya | 1.01 | 0.93 | 0.92 | 3.12 | 3.20% | 98 | -3796 | -9.16% | 41454 |
| Nicaragua | 1.05 | 0.92 | 0.87 | -1.68 | -1.51% | 111 | -1964 | -19.65% | 9999 |
| Bangladesh | 1.07 | 0.9 | 0.84 | -0.35 | -0.32% | 109 | -10188 | -11.79% | 86410 |
| Mexico | 1 | 0.9 | 0.9 | -1.51 | -1.13% | 134 | 0 | 0.00% | 94064 |
| Belarus | 1.05 | 0.88 | 0.84 | -1.45 | -1.11% | 131 | -2978 | -14.42% | 20649 |
| Morocco | 1.02 | 0.88 | 0.86 | -2.44 | -1.75% | 140 | 0 | 0.00% | 14027 |
| Madagascar | 0.96 | 0.87 | 0.91 | 10.84 | 11.44% | 95 | -2401 | -9.92% | 24216 |
| Uruguay | 1.03 | 0.86 | 0.84 | -1.13 | -0.90% | 126 | -9491 | -32.67% | 29047 |
| Azerbaijan | 1.06 | 0.85 | 0.81 | -1.4 | -1.11% | 126 | -355 | -5.14% | 6912 |
| Costa Rica | 1.1 | 0.83 | 0.77 | -0.62 | -0.51% | 120 | -416 | -10.93% | 3808 |
| Peru | 0.99 | 0.83 | 0.84 | 4.02 | 3.49% | 115 | -171 | -0.64% | 26636 |
| Burkina Faso | 1 | 0.82 | 0.81 | -0.52 | -0.42% | 122 | -6842 | -32.47% | 21071 |
| Yemen | 0.96 | 0.79 | 0.82 | 13.75 | 13.89% | 99 | -891 | -11.38% | 7826 |
| South Africa | 0.98 | 0.78 | 0.8 | 6.06 | 4.95% | 123 | 0 | 0.00% | 31956 |
| Colombia | 0.87 | 0.77 | 0.89 | 13 | 10.65% | 122 | -44 | -0.07% | 64412 |
| Mongolia | 1.06 | 0.75 | 0.71 | -0.08 | -0.07% | 107 | -5396 | -24.62% | 21915 |
| Ecuador | 0.93 | 0.72 | 0.77 | 11.47 | 10.55% | 109 | 0 | 0.00% | 14739 |
| Paraguay | 0.96 | 0.71 | 0.75 | 4.12 | 3.59% | 115 | -6696 | -23.16% | 28918 |
| Tajikistan | 1.05 | 0.71 | 0.67 | 7.03 | 7.57% | 93 | -1051 | -18.86% | 5573 |
| Iraq | 0.82 | 0.7 | 0.85 | 33.13 | 32.39% | 102 | 0 | 0.00% | 7530 |
| Kyrgyzstan | 1.08 | 0.69 | 0.65 | 0.5 | 0.44% | 115 | -714 | -15.02% | 4752 |
| Bolivia | 1.04 | 0.67 | 0.65 | 0.41 | 0.39% | 103 | -5299 | -19.79% | 26781 |
| Namibia | 0.85 | 0.66 | 0.8 | 16.74 | 16.39% | 102 | -2151 | -24.26% | 8866 |
| Georgia | 0.84 | 0.64 | 0.77 | 6.91 | 5.74% | 120 | -9 | -0.39% | 2387 |
| Honduras | 0.81 | 0.63 | 0.78 | 10.09 | 8.89% | 114 | 0 | 0.00% | 6793 |
| Senegal | 0.86 | 0.63 | 0.75 | 6.89 | 6.27% | 110 | -1819 | -16.53% | 11005 |
| El Salvador | 0.88 | 0.62 | 0.71 | 9.4 | 8.19% | 115 | 0 | 0.00% | 3054 |
| Guyana | 0.92 | 0.62 | 0.67 | 6.47 | 5.34% | 121 | -1262 | -60.63% | 2082 |
| Zambia | 0.64 | 0.59 | 0.92 | 33.93 | 38.00% | 89 | -1389 | -5.78% | 24033 |
| **Mean/Median (for Score)** | 1.04 | 0.87 | 0.86 | 3 | **2.56%** | 117 | -9950 | -16.53% | 60193 |
| **Total** | - | - | - | - | **-** | - | **-437 795** | - | **2 648 501** |
| **Cluster 3** | **Score** | **MF** | **TGR (MF:SCORE)** | **Slack FS** | **Slack FS %** | **Current level FS** | **Slack GHG** | **Slack GHG %.** | **Current level GHG(Gg)** |
| Bohemia | 1.32 | 1.17 | 0.94 | -11.19 | -10.24% | 109 | -65 | -16.00% | 407 |
| Croatia | 1.24 | 1.06 | 0.92 | -16.19 | -13.38% | 121 | -1398 | -22.06% | 6335 |
| Denmark | 1.07 | 1.06 | 0.99 | 1.17 | 0.88% | 133 | -1135 | -10.65% | 10658 |
| Netherlands | 1.06 | 1.06 | 1 | -0.39 | -0.31% | 125 | -3084 | -15.64% | 19720 |
| Belgium | 1.07 | 1.05 | 0.99 | -2.64 | -1.80% | 147 | -1473 | -14.66% | 10047 |
| Germany | 1.04 | 1.04 | 1 | -0.54 | -0.38% | 141 | -6651 | -10.48% | 63480 |
| Italy | 1.04 | 1.04 | 1 | 0.57 | 0.40% | 142 | -3036 | -8.93% | 33984 |
| Spain | 1.04 | 1.04 | 0.99 | -0.15 | -0.12% | 128 | -2716 | -6.67% | 40731 |
| United Kingdom | 1.05 | 1.04 | 0.99 | -2.34 | -1.73% | 135 | -7501 | -14.57% | 51482 |
| Greece | 1.03 | 1.02 | 0.99 | -10.36 | -7.53% | 138 | -726 | -8.80% | 8251 |
| Luxembourg | 1.08 | 1.02 | 0.95 | -24.48 | -17.92% | 137 | -76 | -11.57% | 659 |
| Ireland | 1.01 | 1.01 | 0.99 | -4.95 | -3.39% | 146 | -5217 | -21.62% | 24132 |
| Romania | 1.06 | 1 | 0.96 | -3.85 | -2.72% | 142 | -2112 | -13.13% | 16085 |
| Austria | 0.99 | 0.98 | 0.99 | -2.23 | -1.53% | 146 | -287 | -3.71% | 7731 |
| France | 1.01 | 0.98 | 0.97 | 0.09 | 0.06% | 141 | -4775 | -6.14% | 77811 |
| Lithuania | 0.98 | 0.98 | 1 | -0.91 | -0.66% | 137 | -1423 | -27.88% | 5104 |
| Poland | 1.01 | 0.96 | 0.95 | -0.36 | -0.27% | 135 | -1833 | -5.80% | 31628 |
| Hungary | 0.97 | 0.93 | 0.96 | -0.35 | -0.28% | 125 | -710 | -10.32% | 6877 |
| Portugal | 0.91 | 0.81 | 0.89 | -0.44 | -0.32% | 140 | -79 | -1.10% | 7190 |
| Sweden | 0.8 | 0.79 | 0.99 | 1.13 | 0.91% | 124 | -367 | -4.99% | 7358 |
| Finland | 0.75 | 0.74 | 0.98 | 0 | 0.00% | 129 | -163 | -2.71% | 6005 |
| Bulgaria | 0.73 | 0.73 | 0.99 | 1.58 | 1.42% | 111 | -356 | -7.22% | 4931 |
| Estonia | 0.75 | 0.72 | 0.96 | 8.16 | 6.54% | 125 | -273 | -15.39% | 1772 |
| Slovakia | 0.7 | 0.67 | 0.96 | 10.73 | 9.57% | 112 | 0 | 0.00% | 2514 |
| Latvia | 0.67 | 0.64 | 0.96 | 0.26 | 0.20% | 128 | 0 | 0.00% | 2595 |
| Slovenia | 0.69 | 0.64 | 0.94 | 2.88 | 2.28% | 127 | 0 | 0.00% | 1706 |
| Cyprus | 0.65 | 0.62 | 0.94 | 12.67 | 10.36% | 122 | 0 | 0.00% | 3015 |
| **Mean/Median (for Scores)** | 1.01 | 0.98 | 0.98 | -1.56 | **-1.19%** | 131 | -1684 | **-10.05%** | 16748 |
| **Total** | - | - | - | - | **-** | - | **-45 456** | **-** | **452 206** |

Note: MF stands for meta frontier, FS – food security, GHG – greenhouse gases emission, TGR – technology gap

Table A2. Agricultural GHG and food security non-radial slacks in input-oriented model (I-O); 2005-2018 yearly averages

| **Cluster 1** | **Score** | **MF** | **TGR (MF:SCORE)** | **Slack FS** | **Slack FS %** | **Current level FS** | **Slack GHG** | **Slack GHG %.** | **Current level GHG** |
| --- | --- | --- | --- | --- | --- | --- | --- | --- | --- |
| USA | 10.3 | 10.3 | 1 | 0.34 | 0.23% | 148 | -9209 | -2.43% | 379283 |
| Ghana | 3.23 | 2.29 | 0.83 | 0.57 | 0.44% | 129 | -71 | -0.77% | 9224 |
| Algeria | 2.24 | 2.16 | 0.96 | 0 | 0.00% | 142 | -148 | -1.33% | 11161 |
| Argentina | 1.85 | 1.82 | 0.99 | 2.4 | 1.81% | 132 | -10171 | -7.72% | 131740 |
| Ukraine | 1.75 | 1.62 | 0.99 | 9.28 | 7.29% | 127 | -176 | -0.59% | 29848 |
| Egypt | 1.74 | 1.56 | 0.92 | 0.08 | 0.06% | 142 | -106 | -0.34% | 31194 |
| Turkey | 1.54 | 1.38 | 0.96 | 2.65 | 1.72% | 154 | -142 | -0.35% | 40969 |
| Israel | 1.5 | 1.31 | 0.95 | 3 | 1.93% | 156 | -15 | -1.06% | 1406 |
| Malawi | 2.89 | 1.31 | 0.7 | 1.48 | 1.26% | 117 | -75 | -1.56% | 4805 |
| Viet Nam | 1.24 | 1.19 | 0.96 | 0.65 | 0.55% | 119 | -1623 | -2.23% | 72725 |
| Philippines | 1.18 | 1.16 | 0.98 | 0.62 | 0.53% | 116 | -3173 | -5.00% | 63416 |
| Russian Federation | 1.15 | 1.14 | 0.99 | 1.34 | 1.00% | 133 | -3154 | -3.22% | 98022 |
| China | 1.11 | 1.11 | 1 | 8.75 | 6.91% | 127 | -59551 | -8.54% | 697231 |
| Thailand | 1.16 | 1.05 | 0.93 | 5.76 | 5.10% | 113 | -7301 | -9.47% | 77133 |
| Japan | 1.16 | 1.05 | 0.92 | 5.62 | 5.02% | 112 | -85 | -0.35% | 24207 |
| North Macedonia | 2.33 | 0.99 | 0.61 | 0.09 | 0.07% | 119 | -9 | -0.64% | 1421 |
| Chile | 1.11 | 0.94 | 0.84 | 4.33 | 3.53% | 123 | -185 | -1.56% | 11845 |
| Côte d'Ivoire | 1.27 | 0.92 | 0.75 | 6.63 | 5.19% | 128 | -27 | -0.43% | 6394 |
| Canada | 0.95 | 0.91 | 0.97 | 13.6 | 9.82% | 139 | -10916 | -19.18% | 56924 |
| Malaysia | 1.18 | 0.91 | 0.8 | 8.1 | 6.69% | 121 | -353 | -2.49% | 14197 |
| Mozambique | 1.02 | 0.8 | 0.8 | 0.95 | 0.93% | 103 | -8613 | -47.60% | 18095 |
| Norway | 1.27 | 0.79 | 0.68 | 0.17 | 0.13% | 136 | -179 | -3.48% | 5139 |
| Switzerland | 1.09 | 0.76 | 0.71 | 0.34 | 0.26% | 133 | -241 | -3.99% | 6048 |
| Jordan | 1.46 | 0.69 | 0.53 | 1.09 | 0.90% | 121 | -66 | -6.02% | 1092 |
| Albania | 1.41 | 0.65 | 0.5 | 0.1 | 0.07% | 131 | -302 | -9.06% | 3337 |
| Sri Lanka | 1.07 | 0.43 | 0.4 | 0.45 | 0.40% | 112 | -612 | -9.18% | 6663 |
| Armenia | 1.28 | 0.39 | 0.35 | 0 | 0.00% | 120 | -14 | -0.87% | 1586 |
| Saudi Arabia | 0.59 | 0.35 | 0.58 | 0.41 | 0.31% | 133 | -370 | -6.34% | 5841 |
| **Mean/Median (for Scores)** | 1.27 | 1.05 | 0.88 | 2.81 | **2.20%** | 128 | -4174 | **-6.45%** | 64677 |
| **Total** | - | - | - | - | **-** | - | **-116885** | **-** | **1810946** |
| **Cluster 2** | **Score** | **MF** | **TGR (MF:SCORE)** | **Slack FS** | **Slack FS %** | **Current level FS** | **Slack GHG (Gg)** | **Slack GHG %.** | **Current level GHG(Gg)** |
| Iceland | 5.56 | 7.53 | 1.88 | 0.06 | 0.05% | 137 | -3 | -0.47% | 640 |
| Indonesia | 3.24 | 2.94 | 0.92 | 0.81 | 0.69% | 118 | -374 | -0.23% | 165286 |
| India | 1.91 | 2.62 | 1.67 | 7.99 | 7.53% | 106 | -59486 | -8.17% | 727678 |
| Niger | 2.55 | 1.88 | 0.76 | 0 | 0.00% | 119 | -2396 | -9.87% | 24277 |
| Brunei Darussalam | 13.97 | 1.85 | 0.93 | 0 | 0.00% | 127 | 0 | -0.14% | 115 |
| Iran (Islamic Republic of) | 1.48 | 1.43 | 1.05 | 5.68 | 4.46% | 127 | -469 | -1.23% | 37981 |
| Pakistan | 1.37 | 1.35 | 0.98 | 2.81 | 2.62% | 107 | -11778 | -7.15% | 164626 |
| Nepal | 2.15 | 1.32 | 0.82 | 0 | 0.00% | 119 | -1470 | -5.80% | 25339 |
| Cameroon | 2.48 | 1.26 | 0.53 | 0.37 | 0.31% | 118 | 0 | 0.00% | 12963 |
| Brazil | 2.55 | 1.17 | 0.59 | 0.04 | 0.03% | 132 | -15362 | -3.03% | 506303 |
| Kazakhstan | 1.95 | 1.14 | 0.71 | 0.98 | 0.75% | 130 | -1831 | -7.47% | 24497 |
| New Zealand | 1.42 | 1.11 | 0.86 | 0.21 | 0.16% | 127 | -1408 | -3.18% | 44318 |
| Tunisia | 1.46 | 1.04 | 0.82 | 0.53 | 0.37% | 142 | -32 | -0.70% | 4633 |
| Ethiopia | 1.12 | 1.03 | 0.93 | 0.46 | 0.46% | 101 | -15283 | -15.43% | 99013 |
| Kenya | 1.11 | 0.93 | 0.87 | 3.58 | 3.66% | 98 | -3119 | -7.52% | 41454 |
| Nicaragua | 1.07 | 0.9 | 0.84 | 0 | 0.00% | 111 | -2155 | -21.56% | 9999 |
| Australia | 2.18 | 0.89 | 0.5 | 0.14 | 0.11% | 132 | -21364 | -13.36% | 159919 |
| Bangladesh | 1.17 | 0.88 | 0.76 | 0.54 | 0.49% | 109 | -844 | -0.98% | 86410 |
| Madagascar | 0.98 | 0.82 | 0.82 | 5.5 | 5.81% | 95 | -3580 | -14.78% | 24216 |
| Mexico | 1.04 | 0.81 | 0.79 | 0 | 0.00% | 134 | -526 | -0.56% | 94064 |
| Belarus | 1.14 | 0.8 | 0.7 | 0 | 0.00% | 131 | -1677 | -8.12% | 20649 |
| Uruguay | 1.09 | 0.77 | 0.71 | 0.64 | 0.51% | 126 | -7435 | -25.60% | 29047 |
| Burkina Faso | 1.03 | 0.74 | 0.71 | 0 | 0.00% | 122 | -8125 | -38.56% | 21071 |
| Yemen | 1.01 | 0.73 | 0.72 | 5.99 | 6.05% | 99 | -858 | -10.97% | 7826 |
| Azerbaijan | 1.33 | 0.73 | 0.6 | 0 | 0.00% | 126 | -68 | -0.98% | 6912 |
| Mongolia | 1.13 | 0.71 | 0.63 | 0.24 | 0.23% | 107 | -5280 | -24.10% | 21915 |
| Peru | 0.99 | 0.68 | 0.69 | 5.03 | 4.36% | 115 | -136 | -0.51% | 26636 |
| Costa Rica | 1.71 | 0.65 | 0.46 | 0.4 | 0.33% | 120 | -6 | -0.17% | 3808 |
| Tajikistan | 1.37 | 0.64 | 0.51 | 5.36 | 5.77% | 93 | -380 | -6.81% | 5573 |
| South Africa | 0.98 | 0.63 | 0.64 | 3.51 | 2.86% | 123 | 0 | 0.00% | 31956 |
| Colombia | 0.78 | 0.61 | 0.78 | 2.93 | 2.40% | 122 | -16077 | -24.96% | 64412 |
| Morocco | 1.34 | 0.57 | 0.45 | 0.02 | 0.01% | 140 | -50 | -0.35% | 14027 |
| Namibia | 0.83 | 0.52 | 0.6 | 16.79 | 16.44% | 102 | -5017 | -56.59% | 8866 |
| Kyrgyzstan | 1.16 | 0.46 | 0.4 | 0.55 | 0.48% | 115 | -102 | -2.15% | 4752 |
| Paraguay | 0.95 | 0.45 | 0.48 | 2.55 | 2.22% | 115 | -11151 | -38.56% | 28918 |
| Iraq | 0.7 | 0.45 | 0.64 | 13.98 | 13.67% | 102 | -1212 | -16.10% | 7530 |
| Zambia | 0.57 | 0.39 | 0.68 | 0 | 0.00% | 89 | -18985 | -78.99% | 24033 |
| Guyana | 0.91 | 0.38 | 0.41 | 0 | 0.00% | 121 | -1433 | -68.82% | 2082 |
| Senegal | 0.82 | 0.36 | 0.43 | 6.43 | 5.85% | 110 | -5664 | -51.47% | 11005 |
| Georgia | 0.72 | 0.34 | 0.47 | 0 | 0.00% | 120 | -756 | -31.69% | 2387 |
| Ecuador | 0.91 | 0.3 | 0.33 | 10.2 | 9.38% | 109 | -401 | -2.72% | 14739 |
| Bolivia | 1.07 | 0.3 | 0.28 | 1.24 | 1.21% | 103 | -4203 | -15.69% | 26781 |
| El Salvador | 0.79 | 0.27 | 0.34 | 0 | 0.00% | 115 | -451 | -14.78% | 3054 |
| Honduras | 0.67 | 0.27 | 0.4 | 2.35 | 2.07% | 114 | -1601 | -23.56% | 6793 |
| **Mean/Median (for Score)** | 1.12 | 0.75 | 0.69 | 2 | **2.10%** | 117 | -5285 | **-8.78%** | 60193 |
| **Total** | - | - | - | - | **-** | - | **-232550** | **-** | **2648501** |
| **Cluster 3** | **Score** | **MF** | **TGR (MF:SCORE)** | **Slack FS** | **Slack FS %** | **Current level FS** | **Slack GHG** | **Slack GHG %.** | **Current level GHG** |
| Czechia | 2.72 | 2.18 | 0.98 | 0 | 0.00% | 109.2143 | -5 | -1.13% | 407 |
| Luxembourg | 5.49 | 1.73 | 0.9 | 0 | 0.00% | 136.5714 | -12 | -1.78% | 659 |
| Spain | 1.26 | 1.39 | 1.15 | 6.14 | 4.81% | 127.5714 | -454 | -1.12% | 40731 |
| Ireland | 1.23 | 1.29 | 1.08 | 0 | 0.00% | 146.2143 | -6264 | -25.96% | 24132 |
| Italy | 1.13 | 1.27 | 1.06 | 0.93 | 0.65% | 142.4286 | -759 | -2.23% | 33984 |
| Romania | 1.53 | 1.17 | 0.83 | 0.1 | 0.07% | 141.6429 | -829 | -5.15% | 16085 |
| Netherlands | 1.19 | 1.17 | 0.99 | 0.31 | 0.25% | 125.2857 | -724 | -3.67% | 19720 |
| Croatia | 1.54 | 1.17 | 0.83 | 0.1 | 0.08% | 121 | -880 | -13.90% | 6335 |
| Denmark | 1.17 | 1.14 | 0.98 | 2.22 | 1.67% | 132.7857 | -135 | -1.26% | 10658 |
| Belgium | 1.73 | 1.14 | 0.77 | 0 | 0.00% | 146.5714 | -106 | -1.05% | 10047 |
| Greece | 1.41 | 1.12 | 0.86 | 0 | 0.00% | 137.6429 | -264 | -3.20% | 8251 |
| Germany | 1.13 | 1.09 | 0.97 | 0.14 | 0.10% | 140.5714 | -997 | -1.57% | 63480 |
| United Kingdom | 1.11 | 1.08 | 0.98 | 0.33 | 0.24% | 135.3571 | -3675 | -7.14% | 51482 |
| Lithuania | 1.04 | 1.01 | 0.98 | 0 | 0.00% | 137 | -1674 | -32.80% | 5104 |
| Austria | 1.18 | 1.01 | 0.89 | 0 | 0.00% | 145.5714 | -205 | -2.65% | 7731 |
| France | 1.04 | 0.96 | 0.92 | 2.42 | 1.72% | 141.0714 | -4431 | -5.69% | 77811 |
| Poland | 1.04 | 0.95 | 0.92 | 0 | 0.00% | 134.9286 | -1264 | -4.00% | 31628 |
| Hungary | 0.94 | 0.87 | 0.92 | 0 | 0.00% | 125.4286 | -1263 | -18.37% | 6877 |
| Sweden | 0.73 | 0.72 | 0.99 | 0 | 0.00% | 124.0714 | -4112 | -55.89% | 7358 |
| Estonia | 0.73 | 0.69 | 0.95 | 0 | 0.00% | 124.7857 | -1177 | -66.45% | 1772 |
| Portugal | 0.81 | 0.67 | 0.83 | 0 | 0.00% | 139.5714 | -1596 | -22.19% | 7190 |
| Finland | 0.59 | 0.58 | 0.98 | 0 | 0.00% | 129.2143 | -4129 | -68.77% | 6005 |
| Slovakia | 0.58 | 0.56 | 0.97 | 0 | 0.00% | 112.1429 | -1472 | -58.56% | 2514 |
| Bulgaria | 0.51 | 0.5 | 0.99 | 0 | 0.00% | 111.0714 | -3826 | -77.60% | 4931 |
| Latvia | 0.5 | 0.46 | 0.91 | 0 | 0.00% | 127.5 | -2023 | -77.97% | 2595 |
| Slovenia | 0.43 | 0.36 | 0.83 | 0 | 0.00% | 126.5 | -1140 | -66.79% | 1706 |
| Cyprus | 0.34 | 0.29 | 0.86 | 0 | 0.00% | 122.3571 | -2221 | -73.66% | 3015 |
| **Mean/Median (for Scores)** | 1.11 | 1.01 | 0.95 | 0.47 | **0.36%** | 131 | -1690 | **-10.09%** | 16748 |
| **Total** | - | - | - | - | **-** | - | **-45635** | **-** | **452206** |

Note: MF stands for meta frontier, FS – food security, GHG – greenhouse gases emission, TGR – technology gap

Table A3. Agricultural GHG and food security non-radial slacks in non-oriented model (N-O); 2005-2018 yearly averages

| **Cluster 1** | **Score** | **MF** | **TGR (MF:SCORE)** | **Slack FS** | **Slack FS %** | **Current level FS** | **Slack GHG (Gg)** | **Slack GHG %.** | **Current level GHG(Gg)** |
| --- | --- | --- | --- | --- | --- | --- | --- | --- | --- |
| Argentina | 1.44 | 1.36 | 0.96 | -7.05 | -5.34% | 132 | -38748 | -29.41% | 131740 |
| USA | 1.27 | 1.23 | 0.99 | -0.48 | -0.32% | 148 | -37322 | -9.84% | 379283 |
| Ghana | 1.3 | 1.16 | 0.95 | -3.85 | -2.99% | 129 | -891 | -9.66% | 9224 |
| Algeria | 1.27 | 1.15 | 0.95 | -7.43 | -5.24% | 142 | -1276 | -11.43% | 11161 |
| China | 1.15 | 1.15 | 1 | -1.17 | -0.92% | 127 | -53590 | -7.69% | 697231 |
| Egypt | 1.17 | 1.14 | 0.99 | -2.28 | -1.61% | 142 | -1852 | -5.94% | 31194 |
| Malawi | 1.18 | 1.13 | 0.96 | -7.3 | -6.23% | 117 | -407 | -8.46% | 4805 |
| Ukraine | 1.12 | 1.1 | 0.98 | 0.35 | 0.28% | 127 | -1285 | -4.31% | 29848 |
| Israel | 1.23 | 1.1 | 0.95 | -5.87 | -3.77% | 156 | -79 | -5.61% | 1406 |
| Philippines | 1.11 | 1.09 | 0.99 | -0.3 | -0.25% | 116 | -7253 | -11.44% | 63416 |
| Turkey | 1.1 | 1.08 | 0.98 | -3.6 | -2.33% | 154 | -716 | -1.75% | 40969 |
| Viet Nam | 1.08 | 1.07 | 0.99 | -1.07 | -0.90% | 119 | -5100 | -7.01% | 72725 |
| Russian Federation | 1.06 | 1.05 | 0.99 | -0.29 | -0.21% | 133 | -6631 | -6.76% | 98022 |
| Japan | 1.08 | 1.03 | 0.96 | 0 | 0.00% | 112 | -1491 | -6.16% | 24207 |
| Thailand | 1.08 | 1.01 | 0.94 | 0.18 | 0.16% | 113 | -12437 | -16.12% | 77133 |
| North Macedonia | 1.25 | 0.98 | 0.84 | -13.37 | -11.23% | 119 | -155 | -10.87% | 1421 |
| Chile | 1.05 | 0.94 | 0.9 | 3.37 | 2.74% | 123 | -636 | -5.37% | 11845 |
| Côte d'Ivoire | 1.06 | 0.93 | 0.88 | -0.22 | -0.17% | 128 | -146 | -2.29% | 6394 |
| Malaysia | 1.04 | 0.91 | 0.88 | 6.69 | 5.53% | 121 | -1036 | -7.30% | 14197 |
| Canada | 0.92 | 0.9 | 0.98 | 14.8 | 10.69% | 139 | -7933 | -13.94% | 56924 |
| Norway | 1.07 | 0.8 | 0.75 | -6.96 | -5.13% | 136 | -676 | -13.15% | 5139 |
| Switzerland | 1.06 | 0.8 | 0.76 | -0.23 | -0.17% | 133 | -512 | -8.47% | 6048 |
| Mozambique | 0.97 | 0.8 | 0.84 | 0.02 | 0.02% | 103 | -6685 | -36.95% | 18095 |
| Jordan | 1.17 | 0.72 | 0.63 | -9.93 | -8.19% | 121 | -136 | -12.49% | 1092 |
| Albania | 1.03 | 0.68 | 0.67 | -4.89 | -3.73% | 131 | -559 | -16.74% | 3337 |
| Sri Lanka | 1.06 | 0.5 | 0.47 | -0.32 | -0.28% | 112 | -843 | -12.65% | 6663 |
| Saudi Arabia | 0.71 | 0.47 | 0.66 | 5.15 | 3.87% | 133 | 0 | 0.00% | 5841 |
| Armenia | 1 | 0.43 | 0.43 | -2.75 | -2.28% | 120 | 0 | 0.00% | 1586 |
| **Mean/Median (for Score)** | 1.08 | 1.02 | 0.95 | -1.74 | **-1.36%** | 128 | -6728 | **-10.40%** | 64677 |
| **Total** | - | - | - | - | **-** | - | **-188396** |  | **1810946** |
| **Cluster 2** | **Score** | **MF** | **TGR (MF:SCORE)** | **Slack FS** | **Slack FS %** | **Current level FS** | **Slack GHG (Gg)** | **Slack GHG %.** | **Current level GHG(Gg)** |
| Indonesia | 1.33 | 1.32 | 1 | -2.56 | -2.16% | 118 | -24809 | -15.01% | 165286 |
| Brunei Darussalam | 1.68 | 1.3 | 0.9 | -18.19 | -14.28% | 127 | -21 | -18.71% | 115 |
| Niger | 1.3 | 1.29 | 0.99 | -1.49 | -1.25% | 119 | -5834 | -24.03% | 24277 |
| India | 1.27 | 1.25 | 0.99 | -0.88 | -0.83% | 106 | -103645 | -14.24% | 727678 |
| Pakistan | 1.2 | 1.18 | 0.98 | 0 | 0.00% | 107 | -37896 | -23.02% | 164626 |
| Iceland | 1.23 | 1.15 | 0.96 | -6.25 | -4.57% | 137 | -117 | -18.31% | 640 |
| Iran (Islamic Republic of) | 1.18 | 1.14 | 0.98 | -0.14 | -0.11% | 127 | -2743 | -7.22% | 37981 |
| Nepal | 1.23 | 1.14 | 0.96 | -2.4 | -2.02% | 119 | -2741 | -10.82% | 25339 |
| Cameroon | 1.13 | 1.11 | 0.98 | -1.76 | -1.49% | 118 | -226 | -1.74% | 12963 |
| Brazil | 1.21 | 1.09 | 0.94 | -0.61 | -0.46% | 132 | -56399 | -11.14% | 506303 |
| New Zealand | 1.16 | 1.06 | 0.96 | -0.53 | -0.42% | 127 | -3922 | -8.85% | 44318 |
| Ethiopia | 1.02 | 0.99 | 0.97 | -0.93 | -0.92% | 101 | -10190 | -10.29% | 99013 |
| Kazakhstan | 1.17 | 0.99 | 0.87 | -2.52 | -1.94% | 130 | -2808 | -11.46% | 24497 |
| Tunisia | 1.08 | 0.95 | 0.89 | -3.65 | -2.56% | 142 | -45 | -0.97% | 4633 |
| Kenya | 1.02 | 0.92 | 0.9 | 2.34 | 2.40% | 98 | -2455 | -5.92% | 41454 |
| Australia | 1.14 | 0.91 | 0.82 | -1.97 | -1.49% | 132 | -18006 | -11.26% | 159919 |
| Nicaragua | 1.06 | 0.91 | 0.85 | -1 | -0.90% | 111 | -2132 | -21.32% | 9999 |
| Bangladesh | 1.09 | 0.87 | 0.8 | -0.4 | -0.37% | 109 | -6259 | -7.24% | 86410 |
| Mexico | 0.99 | 0.85 | 0.86 | -1.87 | -1.40% | 134 | 0 | 0.00% | 94064 |
| Belarus | 1.06 | 0.84 | 0.79 | -1.5 | -1.15% | 131 | -2118 | -10.26% | 20649 |
| Madagascar | 0.94 | 0.82 | 0.86 | 8.73 | 9.22% | 95 | -2318 | -9.57% | 24216 |
| Uruguay | 1.03 | 0.82 | 0.79 | -1.21 | -0.96% | 126 | -9407 | -32.39% | 29047 |
| Azerbaijan | 1.07 | 0.78 | 0.73 | -3.08 | -2.45% | 126 | -48 | -0.69% | 6912 |
| Burkina Faso | 1 | 0.76 | 0.75 | -0.67 | -0.55% | 122 | -7147 | -33.92% | 21071 |
| Peru | 0.99 | 0.75 | 0.76 | 3.75 | 3.25% | 115 | -87 | -0.33% | 26636 |
| Morocco | 1.03 | 0.75 | 0.73 | -3.1 | -2.22% | 140 | 0 | 0.00% | 14027 |
| Costa Rica | 1.14 | 0.73 | 0.66 | -0.72 | -0.60% | 120 | -137 | -3.61% | 3808 |
| Mongolia | 1.08 | 0.71 | 0.67 | -0.39 | -0.36% | 107 | -4749 | -21.67% | 21915 |
| Yemen | 0.96 | 0.71 | 0.73 | 10.98 | 11.10% | 99 | -801 | -10.24% | 7826 |
| South Africa | 0.98 | 0.69 | 0.7 | 5.58 | 4.55% | 123 | -31 | -0.10% | 31956 |
| Colombia | 0.81 | 0.67 | 0.83 | 9.26 | 7.59% | 122 | -3199 | -4.97% | 64412 |
| Tajikistan | 1.09 | 0.63 | 0.58 | 6.58 | 7.08% | 93 | -774 | -13.88% | 5573 |
| Paraguay | 0.95 | 0.58 | 0.61 | 3.5 | 3.04% | 115 | -8696 | -30.07% | 28918 |
| Namibia | 0.81 | 0.52 | 0.63 | 16.91 | 16.56% | 102 | -3861 | -43.54% | 8866 |
| Kyrgyzstan | 1.11 | 0.51 | 0.46 | 0.42 | 0.37% | 115 | -402 | -8.46% | 4752 |
| Iraq | 0.74 | 0.49 | 0.66 | 30.27 | 29.59% | 102 | 0 | 0.00% | 7530 |
| Ecuador | 0.91 | 0.48 | 0.53 | 11 | 10.12% | 109 | 0 | 0.00% | 14739 |
| Bolivia | 1.05 | 0.47 | 0.45 | 0.01 | 0.01% | 103 | -4460 | -16.65% | 26781 |
| Zambia | 0.55 | 0.4 | 0.71 | 34.56 | 38.70% | 89 | -14956 | -62.23% | 24033 |
| Senegal | 0.82 | 0.39 | 0.45 | 6.59 | 5.99% | 110 | -4371 | -39.72% | 11005 |
| Georgia | 0.74 | 0.34 | 0.46 | 4.53 | 3.76% | 120 | -140 | -5.86% | 2387 |
| Guyana | 0.91 | 0.33 | 0.36 | 6.33 | 5.22% | 121 | -1367 | -65.69% | 2082 |
| Honduras | 0.7 | 0.3 | 0.42 | 9.88 | 8.70% | 114 | 0 | 0.00% | 6793 |
| El Salvador | 0.81 | 0.26 | 0.32 | 9.11 | 7.94% | 115 | -35 | -1.15% | 3054 |
| **Mean/Median (for Score)** | 1.06 | 0.8 | 0.79 | 3 | **2.39%** | 117 | -7940 | **-13.19%** | 60193 |
| **Total** |  |  |  |  |  |  | **-349354** |  | **2648501** |
| **Cluster 3** | **Score** | **MF** | **TGR (MF:SCORE)** | **Slack FS** | **Slack FS %** | **Current level FS** | **Slack GHG(Gg)** | **Slack GHG %.** | **Current level GHG(Gg)** |
| Czechia | 1.38 | 1.29 | 0.96 | -5.65 | -5.17% | 109.2143 | -27 | -6.54% | 407 |
| Netherlands | 1.09 | 1.09 | 1 | -0.48 | -0.38% | 125.2857 | -2230 | -11.31% | 19720 |
| Croatia | 1.25 | 1.08 | 0.92 | -14.29 | -11.81% | 121 | -1161 | -18.32% | 6335 |
| Denmark | 1.09 | 1.08 | 0.99 | 0.75 | 0.57% | 132.7857 | -621 | -5.83% | 10658 |
| Belgium | 1.08 | 1.07 | 0.99 | -2.56 | -1.74% | 146.5714 | -864 | -8.60% | 10047 |
| Germany | 1.06 | 1.05 | 0.99 | -0.68 | -0.49% | 140.5714 | -4440 | -6.99% | 63480 |
| Italy | 1.06 | 1.05 | 0.99 | -0.11 | -0.07% | 142.4286 | -1761 | -5.18% | 33984 |
| United Kingdom | 1.07 | 1.05 | 0.98 | -1.93 | -1.43% | 135.3571 | -6194 | -12.03% | 51482 |
| Spain | 1.06 | 1.05 | 0.99 | -0.06 | -0.05% | 127.5714 | -1054 | -2.59% | 40731 |
| Luxembourg | 1.08 | 1.02 | 0.95 | -24.12 | -17.66% | 136.5714 | -73 | -11.16% | 659 |
| Greece | 1.03 | 1.01 | 0.98 | -10.92 | -7.93% | 137.6429 | -623 | -7.55% | 8251 |
| Ireland | 1.01 | 1 | 0.99 | -6.17 | -4.22% | 146.2143 | -5920 | -24.53% | 24132 |
| Romania | 1.08 | 1 | 0.94 | -3.6 | -2.54% | 141.6429 | -1604 | -9.97% | 16085 |
| Lithuania | 0.98 | 0.98 | 1 | -0.91 | -0.66% | 137 | -1528 | -29.95% | 5104 |
| Austria | 0.99 | 0.97 | 0.98 | -2.23 | -1.53% | 145.5714 | -362 | -4.68% | 7731 |
| France | 1.03 | 0.97 | 0.94 | 0.04 | 0.03% | 141.0714 | -4286 | -5.51% | 77811 |
| Poland | 1.01 | 0.96 | 0.94 | -0.29 | -0.22% | 134.9286 | -1442 | -4.56% | 31628 |
| Hungary | 0.95 | 0.9 | 0.95 | -0.3 | -0.24% | 125.4286 | -944 | -13.73% | 6877 |
| Sweden | 0.75 | 0.75 | 0.99 | 2.48 | 2.00% | 124.0714 | -2928 | -39.79% | 7358 |
| Portugal | 0.82 | 0.71 | 0.87 | -0.44 | -0.32% | 139.5714 | -277 | -3.86% | 7190 |
| Estonia | 0.73 | 0.7 | 0.96 | 8.83 | 7.07% | 124.7857 | -956 | -53.95% | 1772 |
| Finland | 0.66 | 0.64 | 0.97 | 0 | 0.00% | 129.2143 | -2850 | -47.46% | 6005 |
| Slovakia | 0.6 | 0.58 | 0.97 | 15.94 | 14.21% | 112.1429 | -736 | -29.28% | 2514 |
| Bulgaria | 0.57 | 0.56 | 0.98 | 1.12 | 1.01% | 111.0714 | -2625 | -53.24% | 4931 |
| Latvia | 0.48 | 0.45 | 0.95 | 1.33 | 1.05% | 127.5 | -1497 | -57.68% | 2595 |
| Slovenia | 0.36 | 0.35 | 0.95 | 3.25 | 2.57% | 126.5 | -434 | -25.44% | 1706 |
| Cyprus | 0.35 | 0.3 | 0.86 | 14.59 | 11.93% | 122.3571 | -767 | -25.43% | 3015 |
| **Mean/Median (for Score)** | 1.01 | 0.98 | 0.97 | -0.98 | **-0.74%** | 131 | -1785 | **-10.66%** | 16748 |
| **Total** | - | - | - | - | - | - | **-48203** | **-** | **452206** |

Note: MF stands for meta frontier, FS – food security, GHG – greenhouse gases emission, TGR – technology gap

Table A4. Metafrontier TFP index and its decomposition in Clusters 1-3 and selected countries (2006-2018 average; output-oriented model).

| **DMU** | MI (t-1, t) | EC (t-1, t) | TC (t-1, t) | OBTC (t-1, t) | IBTC (t-1, t) | MATC (t-1, t) |
| --- | --- | --- | --- | --- | --- | --- |
| **Cluster 1** | | | | | | |
| China | 1.005 | 0.976 | 1.035 | 0.985 | 0.984 | 1.074 |
| USA | 0.983 | 0.959 | 1.042 | 0.990 | 0.978 | 1.098 |
| **Cluster mean** | **1.008** | **0.990** | **1.020** | **0.988** | **1.000** | **1.036** |
| **Cluster 2** | | | | | | |
| India | 1.015 | 0.973 | 1.051 | 0.968 | 0.990 | 1.112 |
| Indonesia | 1.042 | 0.991 | 1.074 | 0.954 | 0.996 | 1.168 |
| **Cluster mean** | **1.012** | **0.995** | **1.018** | **0.990** | **1.002** | **1.030** |
| **Cluster 3** | | | | | | |
| Bohemia | 0.988 | 0.956 | 1.051 | 0.970 | 0.991 | 1.117 |
| France | 1.001 | 0.994 | 1.007 | 0.999 | 1.003 | 1.004 |
| Germany | 1.000 | 0.984 | 1.017 | 0.990 | 0.996 | 1.033 |
| Italy | 0.999 | 0.983 | 1.019 | 0.990 | 0.994 | 1.039 |
| Netherlands | 1.007 | 0.986 | 1.023 | 0.983 | 0.996 | 1.047 |
| Poland | 1.012 | 1.000 | 1.013 | 1.001 | 1.000 | 1.012 |
| Spain | 1.019 | 1.002 | 1.017 | 0.986 | 0.999 | 1.034 |
| United Kingdom | 1.000 | 0.981 | 1.021 | 0.988 | 0.993 | 1.042 |
| **Cluster mean** | **1.006** | **0.993** | **1.014** | **0.993** | **0.999** | **1.024** |

Note: MI - Malmquist index (sequential), EC - efficiency change, TC - technical change, OBTC - output-based technological change, IBTC - input-based technological change, MATC - mixed technological change.
